# LPA Induces Colon Cancer Cell Proliferation through a Cooperation between the ROCK and STAT-3 Pathways

**DOI:** 10.1371/journal.pone.0139094

**Published:** 2015-09-29

**Authors:** Fernanda Leve, Rubem J. Peres-Moreira, Renata Binato, Eliana Abdelhay, José A. Morgado-Díaz

**Affiliations:** 1 Diretoria de Metrologia Aplicada às Ciências da Vida–Dimav, Instituto Nacional de Metrologia, Qualidade e Tecnologia–INMETRO, Xerém, Duque de Caxias, RJ, Brasil; 2 Grupo de Biologia Estrutural, Divisão de Biologia Celular, Centro de Pesquisas, Instituto Nacional de Câncer—INCA, Rio de Janeiro, RJ, Brasil; 3 Centro de Transplante de Medula Óssea (CEMO), Instituto Nacional de Câncer—INCA, Rio de Janeiro, RJ, Brasil; University of Kansas School of Medicine, UNITED STATES

## Abstract

Lysophosphatidic acid (LPA) plays a critical role in the proliferation and migration of colon cancer cells; however, the downstream signaling events underlying these processes remain poorly characterized. The aim of this study was to investigate the signaling pathways triggered by LPA to regulate the mechanisms involved in the progression of colorectal cancer (CRC). We have used three cell line models of CRC, and initially analyzed the expression profile of LPA receptors (LPAR). Then, we treated the cells with LPA and events related to their tumorigenic potential, such as migration, invasion, anchorage-independent growth, proliferation as well as apoptosis and cell cycle were evaluated. We used the Chip array technique to analyze the global gene expression profiling that occurs after LPA treatment, and we identified cell signaling pathways related to the cell cycle. The inhibition of these pathways verified the conclusions of the transcriptomic analysis. We found that the cell lines expressed LPAR1, -2 and -3 in a differential manner and that 10 μM LPA did not affect cell migration, invasion and anchorage-independent growth, but it did induce proliferation and cell cycle progression in HCT-116 cells. Although LPA in this concentration did not induce transcriptional activity of β-catenin, it promoted the activation of Rho and STAT-3. Moreover, ROCK and STAT-3 inhibitors prevented LPA-induced proliferation, but ROCK inhibition did not prevent STAT-3 activation. Finally, we observed that LPA regulates the expression of genes related to the cell cycle and that the combined inhibition of ROCK and STAT-3 prevented cell cycle progression and increased the LPA-induced expression of cyclins E1, A2 and B1 to a greater degree than either inhibitor alone. Overall, these results demonstrate that LPA increases the proliferative potential of colon adenocarcinoma HCT-116 cells through a mechanism involving cooperation between the Rho-ROCK and STAT3 pathways involved in cell cycle control.

## Introduction

Lysophosphatidic acid (LPA) is a naturally occurring bioactive lysophospholipid present in most tissues and biological fluids. LPA can be generated by both lysophospholipase D (lyso-PLD), such as autotaxin (ATX), or via phospholipase A1 or A2 (PLA1 and PLA2, respectively) [1]. ATX was first identified in malignant melanoma as a chemotactic factor necessary for melanoma invasiveness [2], and ATX/Lyso-PLD are aberrantly expressed in many human cancers and in inflammatory bowel disease [1,3]. Moreover, high levels of LPA were found in the plasma and ascitic fluid of ovarian cancer patients [4]; likewise, high levels of lysophosphatidylcholine (LPC), an LPA precursor, were found in the plasma of colorectal cancer (CRC) patients [5]. Although the increase in LPA levels in fluids from patients with CRC has not yet been directly demonstrated, Lin *et al*. [6] have shown that the oral administration of LPA to Apc^Min/+^ mice, which is a model widely used in CRC, nearly doubled the number of polyps in the intestine. Together, these studies support the notion that LPA plays an important role in CRC pathology, which is the third most frequent cancer in men and the second most frequent in women worldwide [7].

Via its binding to specific G-protein-coupled receptors (GPCRs), LPA mediates many biological responses in cancer. In CRC cells, LPA increases proliferation [8], apoptosis protection [9], migration [10] and adaptation to hypoxia [11]. It is well accepted that the cellular response to LPA depends on the expression pattern of LPA receptors (LPARs) because it varies widely among different tissues and cell types. There are currently six recognized LPARs, LPA_1-6_, that are overexpressed in different types of cancers, including CRC [12,13,14]. Among these LPARs, the classical well-known LPARs, LPA_1–3_, belong to the endothelial cell differentiation gene (EDG) family of GPCRs and have been described to regulate different behaviors in CRC cells. For example, by using specific RNA interference, it was shown that LPA_2_ and LPA_3_ but not LPA_1_ are targets for the LPA-induced proliferation of HCT-116 and LS174T [8]. Additionally, it was shown that LPA_1_ mediates the LPA-stimulated cell scattering of DLD-1 cells using a shRNA-lentivirus system [15]. Moreover, LPA_3_ knockdown increases the migration and invasion of HCT-116 cells [16].

LPARs trigger a range of downstream signaling pathways. It is well-established that LPA produces Rho-dependent cytoskeletal responses such as stress fiber formation [17], which are structures related to cell migration. Indeed, we showed that in Caco-2 cells, LPA-induced cell migration is Rho-ROCK dependent [10]. Additionally, Rho-ROCK signaling was also reported to play a role in the regulation of cell proliferation [18]. Although some studies have already shown that LPA stimulates the proliferation of colon cancer cells such as HCT-116 and SW480 [8,19], the Rho-ROCK signaling participation in this event was not addressed.

The signal transducer and activator of transcription 3 (STAT-3) is a transcription factor involved in tumorigenesis processes. Constitutive STAT-3 activation is associated with various human cancers and commonly suggests poor prognosis [20]. It was previously shown that ROCK stimulates STAT-3 activation through Janus Kinase 1 (JAK 1); STAT-3 and JAK 1 cooperate to control actomyosin contractility to mediate the rounded amoeboid migration in melanoma cells [21]. Additionally, it was observed that LPA induces cell motility through STAT-3 phosphorylation in ovarian cancer cells [22]. Interestingly, STAT-3 phosphorylation is implicated in HCT-116 colon cancer cell growth [23]. Nevertheless, it is unknown whether LPA activates STAT-3 in CRC cells or causes the activation of Rho-ROCK participation in this signaling pathway.

Thus, the aim of this study was to analyze the role that LPA plays in different biological processes of CRC progression, such as migration, invasion and proliferation, and to determine the mechanisms underlying these events. Here, we demonstrated that LPA increases HCT-116 cell proliferation through a cascade that integrates RhoA-ROCK and STAT-3 signaling to control cyclin expression and cell cycle progression.

## Materials and Methods

### Antibodies and reagents

L-α-lysophosphatidic acid (oleoyl sodium cat. no. L7260), rabbit anti-LPA_1_ (N-terminal; cat. No. SAB4500689), rabbit anti-LPA_2_ (cat. no. HPA019616), rabbit anti-LPA_3_ (cat. no. HPA013421), anti-Cyclin B1 (cat. no. SAB4503501), 40,6-diamidino-2-phenylindole dihydrochloride (DAPI; Cat. no. 32670) and horseradish peroxidase-conjugated goat anti-rabbit and anti-mouse IgG were obtained from Sigma-Aldrich (Saint Louis, MO, USA). Rabbit monoclonal anti-STAT-3 (cat. no. 9139), mouse monoclonal anti-phospho-STAT3 (pTyr 705; cat. no. 9131), rabbit polyclonal anti-α tubulin (cat. no. 2144), rabbit monoclonal anti-GSK-3β (cat no. 9315), anti-phospho-GSK-3β (pSer9; cat. no. 9336), mouse monoclonal anti-β-catenin (cat. no. 9582) and anti-GAPDH (cat. no. 2118) were purchased from Cell Signaling Technology (Danvers, MA, USA). STA-21 ((S)-Ochromycinone deoxytetrangomycin; cat. no. sc-200757), anti-Cyclin A2 (cat. no. sc-596) and mouse anti-Cyclin E1(cat. no. sc-247) were purchased from Santa Cruz Biotechnology (Dallas, TX, USA). The Alexa 488-conjugated secondary antibody (cat. no. A11008) was obtained from Molecular Probes (Eugene, OR, USA). Y-27632 ((R)-(+)-trans-N-(4-Pyridyl)-4-(1-aminoethyl)-cyclohexanecarboxamide; cat no. US1688000) was purchased from Calbiochem EMD Millipore (Darmstadt, Germany).

### Cell culture and LPA treatments

The human colorectal adenocarcinoma cell lines Caco-2 (HTB-37TM) and HT-29 (HTB-38TM), the human colorectal carcinoma cell line HCT-116 (CCL-247TM) and the ovarian cancer cell line Ovcar-3 (HTB-161) were obtained from the American Type Culture Collection (ATCC; Manassas, VA, USA). The colon cancer cells were grown in Dulbecco's modified Eagle medium (DMEM) (Invitrogen) supplemented with 10% fetal bovine serum (FBS), penicillin G (60 mg/L) and streptomycin (100 mg/L) at 37°C in a humidified atmosphere of 5% CO_2_/air. The cells were passaged weekly with 0.05% trypsin/0.02% EDTA in PBS solution. The ovarian adenocarcinoma cells were grown in Roswell Park Memorial Institute medium (RPMI 1640) (Sigma-Aldrich) that was supplemented with 20% FBS, penicillin G (60 mg/L) and streptomycin (100 mg/L) at 37°C in a humidified atmosphere of 5% CO_2_/air. Caco-2 cells have a differentiated phenotype when they form a confluent monolayer, with low invasive and metastatic potential; the HT-29 cells are moderately differentiated, while HCT-116 cells have an undifferentiated phenotype and high tumorigenic potential. So, these cells represent different stages of CRC progression. The cell cultures were switched to serum-free medium for 24 h prior to the 10 μM LPA treatment, as previously reported [10].

Selective pharmacological inhibitors were added to the cell cultures 1 h before the LPA treatment and were present throughout the treatment as indicated. The inhibitors were diluted in DMSO and stored at –20°C. Each concentrated solution was diluted immediately before use to give final concentrations of 10 μM Y-27632 (ROCK) and 10 μM STA-21 (STAT-3).

### Western-blot analysis

The treated cells were homogenized in lysis buffer (1% Triton X-100, 0.5% sodium deoxycholate, 0.2% SDS, 150 mM NaCl, 2 mM EDTA, 10 mM Hepes, pH 7.4) containing 20 mM NaF, 1 mM orthovanadate, and a protease inhibitor cocktail (Sigma, MO, 1:100 dilution) for 30 min at 4°C. The homogenized lysates were submitted to centrifugation at 10,000 g for 10 min at 4°C. The supernatants were collected and stored at -80°C for the subsequent analysis. Equal amounts of protein (30 to 60 μg/lane), quantified by the BCA protein assay kit (BioRad, Hercules, CA, USA), were prepared by boiling after the addition of the denaturing sample buffer; they were electrophoretically separated by SDS-PAGE on 7.5, 10 or 12% gels and transferred to nitrocellulose membranes using a semi-dry transfer cell (BioRad) at 10 V for 60 min. The membranes were blocked for 1 h with TBS-T (20 mM Tris-HCl, pH 7.6, 137 mM NaCl and 0.1% Tween-20) containing 5% low-fat dried milk or with 1% BSA (Sigma); they were incubated overnight with the following primary antibodies: anti-LPA_1_ (1:500), anti-LPA_2_ (1:500), anti-LPA_3_ (1:500), anti-α-tubulin (1:1000), anti-GAPDH (1:3000), anti-β-catenin (1:1000), anti-GSK3-β (1:1000), anti-phospho-GSK3-β (Ser9) (1:1000), anti-STAT-3 (1:1000), anti-phospho-STAT-3 (Tyr 705) (1:1000), anti-cyclin A2 (1:2000), anti-cyclin E1 (1:2000) and anti-cyclin B1 (1:2000). After washing, the membranes were incubated for 1 h with peroxidase-conjugated goat anti-rabbit or anti-mouse IgG (1:5000). Then, the membranes were washed, and the protein bands were visualized using an enhanced chemiluminescence kit (GE Healthcare UK Limited, Buckinghamshire, UK). The band images of three independent experiments were quantified by optical density using LabWorks 4.6 software (Bio-Rad Laboratories, Hercules, CA).

### RhoA activation assay

The activities of the Rho protein were determined using a specific G-LISA^TM^ *RhoA Activation Assay Biochem Kit* ^TM^ (Cytoskeleton, CO, EUA) following the manufacturer's instructions. Briefly, Rhotekin RBD bound to the plates was used to precipitate GTP-bound Rho from the cell lysate. The active RhoA was detected using a specific antibody against Rho and was visualized using a chemiluminescence reaction. The level of activation was measured with the absorbance set at 490 nm in a microplate spectrophotometer.

### Wound-healing assay

Cell monolayers were serum-starved for 24 h, treated with LPA and scratched using a sterile pipette tip. For each dish, three wounds were manually made, and the three regular wound sites were verified under an Axio Observer Z1 microscope (Carl Zeiss, Inc., Jena, Germany) equipped with an Axio Cam HRc and an Axio Vision Release 8.2 Image Analyzer; the wounds were then selected and marked. After washing with PBS, fresh media containing the inhibitors was added to the cells, which were incubated at 37°C in serum-free DMEM containing 10 μM LPA. Untreated and treated cells were permitted to migrate into the wounded area and were photographed both immediately after wounding (0 h) and 24 h after wounding. The distance between the two edges of the injury was quantified using Adobe Photoshop 6.0 from three independent experiments. The values are represented as percentages and plotted on the graph.

### Invasion assay

To test tumor cell invasion, a transwell with a 6.5 mm polycarbonate filter (8 μm pore size, Cat. no. 3422; Costar, Cambridge, MA) was coated with 20 μl of Matrigel® (Cat. no. 356230; BD Biosciences, San Diego, CA) diluted in DMEM (1:10) and incubated at 37°C for 30 min. Caco-2 (2,5 x 10^4^), HT-29 (2 x 10^4^), HCT-116 (2 x 10^4^) and Ovcar-3 (2 x 10^4^) cells, in 200 μl of serum-free medium with LPA, were seeded in the upper chamber of the transwell. Culture medium with 20% FBS was added as a chemoattractant in the lower chamber. After 48 h of incubation, the upper surface of the membrane was scrubbed with a cotton swab. The invaded cells in the lower membrane were fixed in ethanol for 10 min and stained with crystal violet. The number of invaded untreated and treated cells was expressed as the average of four random fields under the microscope. The values are represented as percentages and plotted on the graph. Ovcar-3 was used as a positive control of LPA efficacy.

### Anchorage-independent growth

Caco-2, HT-29 and HCT-116 cells were seeded in a 12-well plate (previously covered with 1 ml of 0.6% agarose semi-solid) at a density of 250 cells/well in a solution of DMEM containing 10% SFB and 0.3% agarose for 30 min. DMEM with 10% FBS that either contained LPA or did not contain LPA (control) was added to the top of the semi-solid solution and was renewed every 3 days. After 14 days, the colonies formed were imaged and counted using an Axio Observer Z1 (Carl Zeiss, Inc.) microscope equipped with an Axiocam MRc5 camera.

### Cell viability analysis

HT-29 (1x10^3^ cell/mL) and HCT-116 (2 x 10^4^ cell/mL) cells were seeded and cultured in 96-well plates and, after FBS depletion, treated with one of the following solutions: 10 μM LPA alone, 10 μM STA-21, 10 μM Y-27632, or LPA in combination with these inhibitors for the indicated times before incubation with MTT (Sigma Chemical Co.). The cells were maintained for 2 h at 37°C and centrifuged at 1,500 g for 5 min. The supernatant was removed, and the crystals were dissolved in DMSO. The absorbance at 538 nm was measured with a Spectra Max 190 Spectrophotometer (Molecular Devices Sunnyvale, CA).

### Cell proliferation assay

The crystal violet method was used to measure cell proliferation. Caco-2 (2x10^4^ cell/mL), HT-29 (10^3^ cell/mL) and HCT-116 (2x10^4^ cell/mL) cells were cultured in 96-well plates and were FBS-depleted and treated as indicated with LPA and inhibitors of STAT-3 and ROCK for 24, 48 and 72 h and fixed with ethanol for 10 min. The crystal violet solution (0.05% crystal violet and 20% methanol) was added for 10 min. The cells were washed twice with water and then solubilized with methanol. The absorbance at 595 nm was measured with a Spectra Max 190 spectrophotometer (Molecular Devices, Sunnyvale, CA, USA). The values are represented as percentages and plotted on the graph.

### Apoptosis and survival analysis

HT-29 and HCT-116 cells (10^5^−10^6^ cells/mL) were cultured in six-well microtiter plates and were serum-starved for 24 h. After, they were treated for 24, 48 and 72 h with 10 μM LPA. Apoptosis was detected by an Annexin V/propidium iodide (PI) staining assay to detect early apoptotic cells, late apoptotic cells, and non-apoptotic cells at the indicated times. The cells were washed in ice-cold PBS and resuspended in 100 μL of Annexin V binding buffer (0.1 M Hepes/NaOH (pH 7.4), 1.4 M NaCl, 25 mM CaCl_2_) containing annexin V-FITC and PI (1 μg/mL) for 15 min. A FACS analysis was performed using a FACSCalibur flow cytometer and CellQuest software (BDBiosciences, San Jose, CA, USA); the cells negative for both annexin V and PI were considered viable (survival).

### Cell cycle analysis

HCT-116 cells (10^5^−10^6^ cell/mL) cultured in six-well microtiter plates were FBS depleted and treated for 8, 12 and 16 h with LPA and/or inhibitors of STAT-3 or ROCK. After this period, the cells were harvested by trypsinization and washed once with ice-cold PBS. The cells were then stained in the dark with 75 μM propidium iodide (Sigma) for 10 min in a buffer containing 3.4 mM Tris-HCl (pH 7.6), 10 mM NaCl, 0.2% Triton X-100 and 3500 U/L RNAse. An analysis of the DNA content was performed by collecting 10,000 events for the cell cycle and sub-G1 analysis using a FACSCalibur flow cytometer (BD Transduction Labs, Lexington, KY, EUA) and Mod Fit LT software.

### Immunofluorescence

The cells were plated on coverslips that had been placed on 24-well plates in advance. After FBS depletion and treatment, the cells were washed in PBS supplemented with 100 mM CaCl_2_ and 100 mM MgCl_2_ (PBS/CM) and fixed in 100% methanol for 20 min. The samples were permeabilized with 0.5% TX-100 in PBS for 10 min. Later, the cells were incubated in 50 mM NHCl_4_ in PBS for 10 min and blocked in 3% BSA for 1 h. The cells were incubated with the primary antibodies, anti-β-catenin (1:250) and anti-STAT3 (1:50) for 1 h, followed by an additional hour of incubation with secondary Alexa 488-conjugated anti-rabbit or anti-mouse antibodies. Then, the cells were incubated with 40,6-diamidino-2-phenylindole dihydrochloride (DAPI) (1:1000) for 3 min. The coverslips were washed in PBS and mounted with *n*-propyl-gallate, and cell staining was detected using an Axio Observer Z1 immuno-fluorescence microscope equipped with an Axiocam HRc Rev. 3 camera and an Axiovision Release 4.8.1 image analyzer program (Carl Zeiss Inc., Germany).

### Luciferase assay for TCF activity

Two different TCF luciferase reporter genes were used in this assay: an intact wild-type TCF-luciferase reporter construct (SUPER 8 TOPFLASH) and a mutated TCF-luciferase reporter construct (SUPER 8 FOPFLASH), which was used as a negative control. HCT-116 (2x10^4^) cells were seeded in six-well plates and transiently transfected with 2 μg of the SUPER 8 TOPFLASH or FOPFLASH reporter plasmid, along with 3 μl of the FUGENE® 6 Transfection Reagent (Roche). As a control for transfection efficiency, 0.2 μg of a Renila luciferase construct was included in each transfection. After 24 h of transfection, the cells were washed twice with PBS, FBS depleted for 24h and then treated with 10 μM LPA. The cells were harvested 6 and 24 h after transfection, and extracts were prepared with 200 μl of reporter lysis buffer (Promega). Renila and luciferase activity were assayed according to the manufacturer’s protocol using a Kit Dual-Luciferase Reporter Assay System (Promega). The luciferase activity in each well was normalized to the renila activity. Three independent experiments, each assayed in triplicate, were performed on separate cell passages.

### Expression Chip array data analysis

Total RNA from FBS-depleted HCT-116 cells that were either treated or not with LPA for 12h were obtained using an RNeasy Mini Kit (QIAGEN, USA) according to the manufacturer's instructions. One hundred nanograms of total RNA was used to synthesize the biotinylated cRNA according to the GeneChip whole transcription (WT) sense target-labeling assay (*Affymetrix*, *USA*). After, the biotinylated cRNA was hybridized to the GeneChip human gene 1.0 ST array (*Affymetrix*, *USA*), washed and stained according to the manufacturer’s protocols. The GeneChip arrays were scanned using a GeneChip® Scanner 3000. The Affymetrix Expression Console Software Version 1.0 was used to create summarized expression values (CHP-files), and the Robust Multichip Analysis (RMA) algorithm was applied. The data were analyzed using Partek^®^ software (http://www.partek.com) [24]; differentially expressed genes with ≥ 2-fold-change were used as the criteria to define overexpression or down regulation. The pathway analysis and related processes were obtained using MetaCore^TM^ software (http://thomsonreuters.com/metacore) and Ingenuity^®^ Pathway analysis software (http://www.ingenuity.com).

### Statistical analysis

The statistical analysis of three independent experiments was performed using GraphPad Prism 5.0 (GraphPad Software, San Diego, CA, USA). Statistical analyses of migration, invasion, anchorage-independent growth, TCF activity and protein optical density were performed using Student’s t-tests; the cell cycle analysis was performed using a one-way ANOVA with the Bonferroni post-test; and a two-tailed ANOVA with the Bonferroni post-test was performed for the cell proliferation assay. The data were expressed as the mean ± SEM. A difference was considered statistically significant when *P<0.05; **P<0.01; ***P<0.001.

## Results

### Colorectal cancer cells express LPA receptors in a differential manner, but LPA treatment does not alter cell migration, invasiveness, or anchorage-independent growth

**Fig 1:**
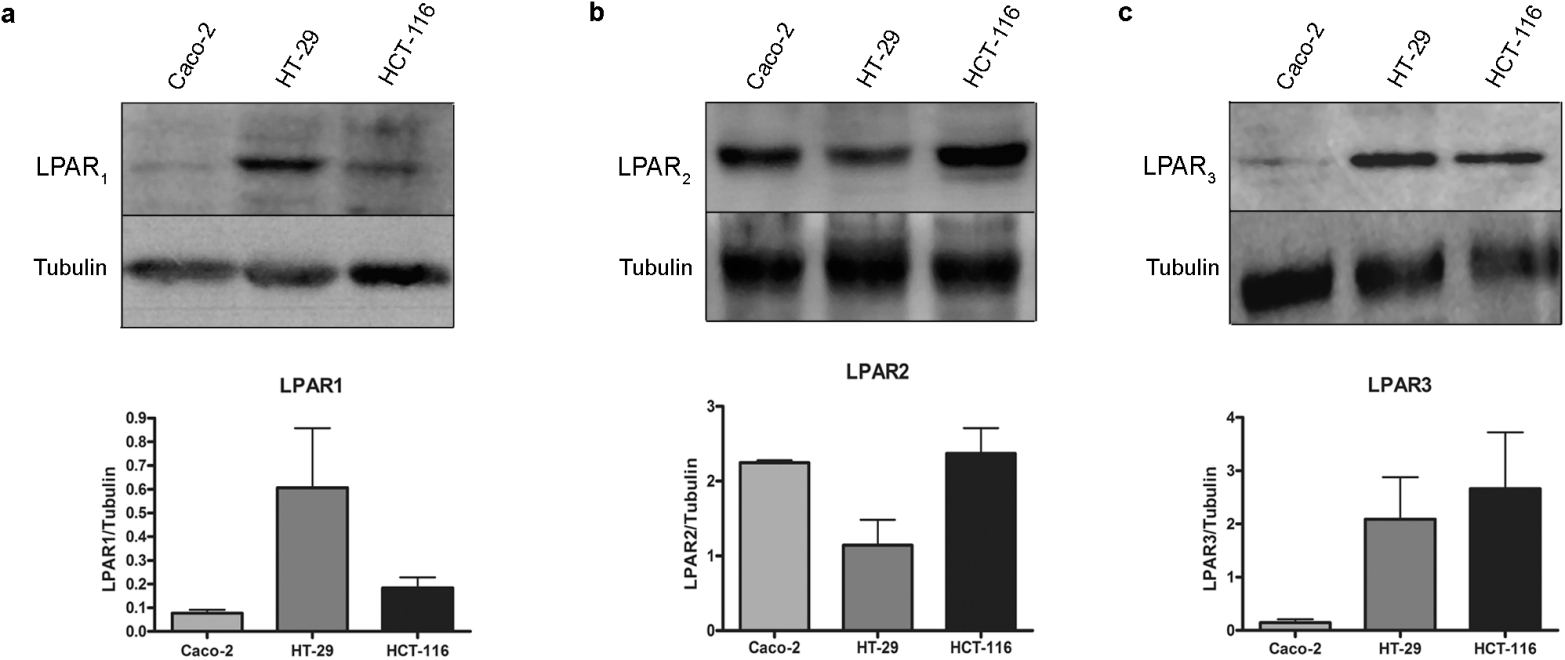
Colon cancer cells present different LPA receptor expression profiles. Representative western blotting and densitometric analyses of Caco-2, HT-29 and HCT-116 cells using specific antibodies against LPAR_1-3_ (a-c, respectively). Caco-2 cells express high levels of LPA_2_, HT-29 cells express high levels of LPA_1-3_ and HCT-116 cells express high levels of LPA_2-3_. The average scores ± SEM. for three independent experiments are shown.

Initially, we investigated the expression levels of LPA receptors in three cell lines of colon cancer, Caco-2, HT-29 and HCT-116, using western blotting. **Fig 1** shows that Caco-2, HT-29 and HCT-116 cells express the receptors LPA_1_, LPA_2_ and LPA_3_ in differential manners. The low-grade invasive Caco-2 cells presented lower levels of LPA_1_ and LPA_3_ in relation to the other cell lines and higher levels of LPA_2_ in relation to HT-29. The intermediately invasive HT-29 cells expressed similar levels of all three LPA receptors; and the highly invasive HCT-116 cells presented higher levels of the receptors LPA_2_ and LPA_3_, in comparison to Caco-2 and HT-29 cells, but lower levels of LPA_1_ in relation to HT-29. This result indicates that in fact, the three cell lines are responsive to this biolipid. Some studies have shown that LPA mediates cell migration in a wide range of cancer cell types [25,26], and we have previously shown that LPA increases the migration of Caco-2 cells [10]. Thus, we wanted to evaluate the effects of LPA on events related to cancer progression in colon cancer cells with a higher invasive potential. Therefore, we performed wound-healing, a cell invasion assay and anchorage-independent growth (AIG) in Caco-2, HT-29 and HCT-116 cells. We found that LPA did not alter cell migration in HT-29 and HCT-116 cells or increase the invasive potential of the three colon cancer cell lines; moreover, LPA did not alter the tumorigenicity, as evaluated by AIG (**S1, S2 and S3 Figs**, respectively).

### LPA does not induce cell death but increases proliferation by promoting cell progression to the S phase and then the G2/M phases

**Fig 2:**
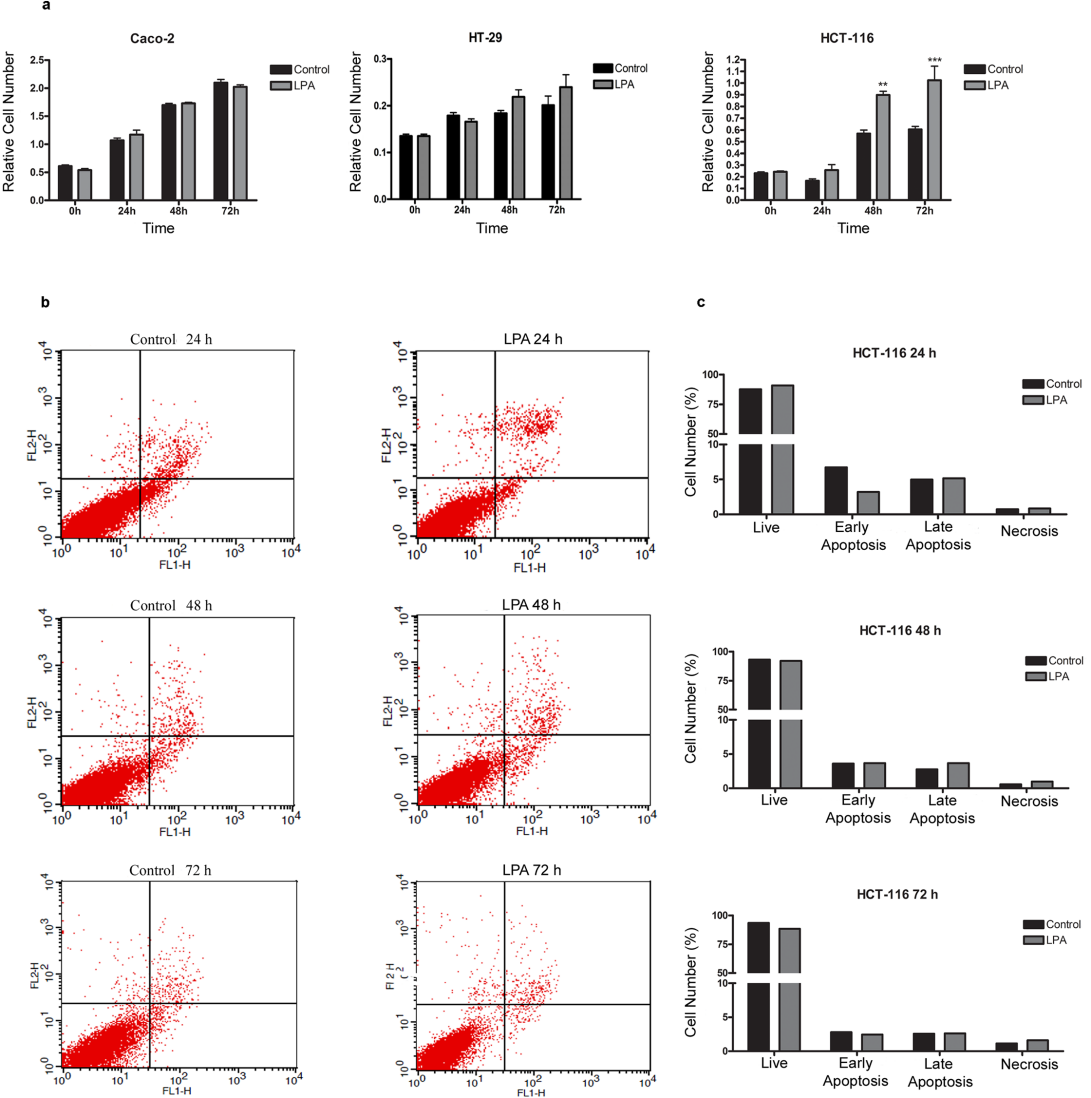
LPA increased proliferation in HCT-116 cells but did not induce cell death. Colon cancer cells were FBS starved for 24 h and then treated with LPA (10 μM) for 24, 48 or 72 h. The relative cell number was evaluated through crystal violet staining (a), and apoptosis was followed by the Annexin-V/PI double staining method (b). a) LPA increased the relative number of HCT-116 cells, but not Caco-2 or HT-29 cells. Statistical analyses were performed using *two-way* ANOVA with *post-hoc* Bonferroni test. ** p< 0.01; *** p<0.001. Average scores± SEM. for three independent experiments are shown. b) FACs analysis via Annexin V-FITC/PI staining showed that LPA did not reduce cell death in HCT-116 cells. Four different cell populations were detected after the Annexin V/PI staining of HCT-116 cells. Alive cells are grouped in the lower left part of the panel, early apoptotic cells are grouped in the lower right part of the panel, late apoptotic cells are grouped in the higher right part of the panel and necrotic cells are grouped in the higher left part of the panel. FL1-H, Annexin V; FL2-H, PI. c) Data obtained from the flow cytometric analyses are plotted in a graph. There was no increased percentage of LPA-mediated apoptosis.

**Fig 3:**
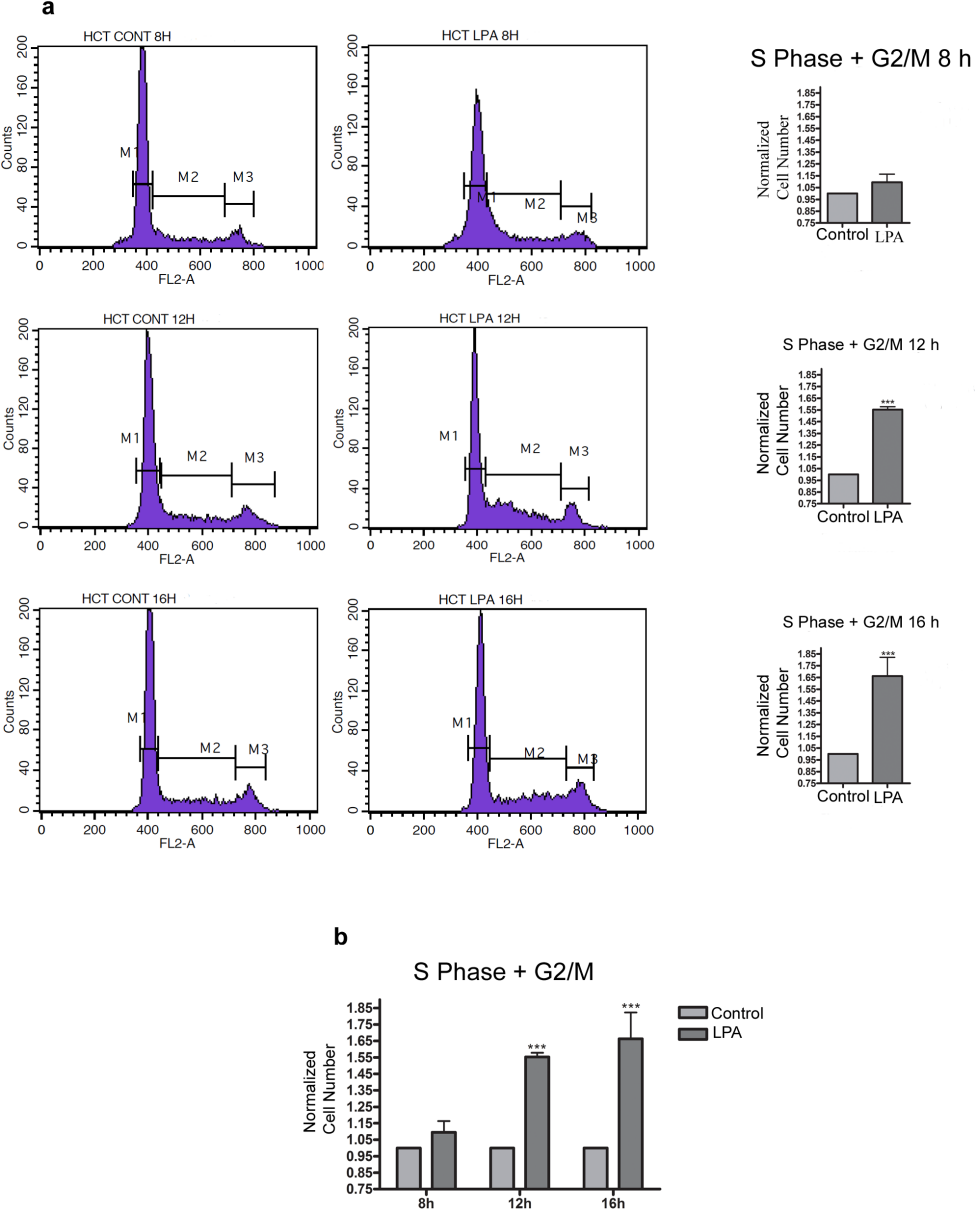
Effect of LPA on cell cycle progression in HCT-116 cells. The cells were FBS starved for 24 h (cont, control), treated with LPA at the indicated times, stained with PI and analyzed using FACS. a) The proportion of cells in the G2/M phase was significantly increased after 12 and 16 h with LPA treatment. M1: cells in G1; M2: cells in the S phase; M3: cells in G2/M. b) The graph indicates the percentage of S and G2/M cells in relation to the control group. The proportion of DNA in the S phase was calculated using ModfitLT Software. Data are shown as the mean ± SEM from three independent experiments. Statistical analyses were performed using *one-way* ANOVA (*** p<0.001). PI, propidium iodide; FACS, fluorescence-activated cell sorting.

Because LPA increases cell proliferation and the evasion of death in colon cancer cells [8,9,27], we decided to evaluate whether LPA modulates cell viability in our study. Our results indicated that after 48 and 72 h of treatment, LPA increased the number of viable HCT-116 cells but not the number of viable Caco-2 and HT-29 cells (**Fig 2A**). Because increased numbers of viable cells can indicate the induction of proliferation or decreased cell death by apoptosis evasion, we determined whether LPA could affect cell death by incubating serum-starved HCT-116 cells with LPA for 24, 48 and 72 h and then staining the cells with Annexin V and PI (**Fig 2B and 2C**). LPA did not alter the number of dead cells, indicating that the increase in cell number observed at **Fig 2A** occurs through the increase in cell proliferation and not the reduction of death. To determine whether the LPA-induced HCT-116 cell growth was a result of the alteration of cell cycle regulation, the cell cycle profiles were monitored by a flow cytometric analysis of DNA content. As shown in **Fig 3**, the distribution in the phases of the cell cycle indicated that treatment with LPA for 8, 12 and 16 h promoted cell progression to the S phase and then the G2/M phases, during which the population increased compared to the serum-starved cells.

### LPA-induced cell proliferation of HCT-116 involves Rho-ROCK signaling activation

Based on previous studies showing that LPA activates the small GTPase Rho [14] and that Rho regulates cell proliferation in different cell types [28], we decided to investigate whether LPA-inducing proliferation of HCT-116 cells is Rho-ROCK dependent. The cell layers were serum starved for 1 h, followed by treatment with 10 μM LPA at the indicated times; we then performed the Rho activity assay. **S4 Fig** shows that LPA induces RhoA activation primarily at 5 and 15 minutes of treatment. This result corroborates previous studies showing that LPA activates RhoA GTPase.

**Fig 4:**
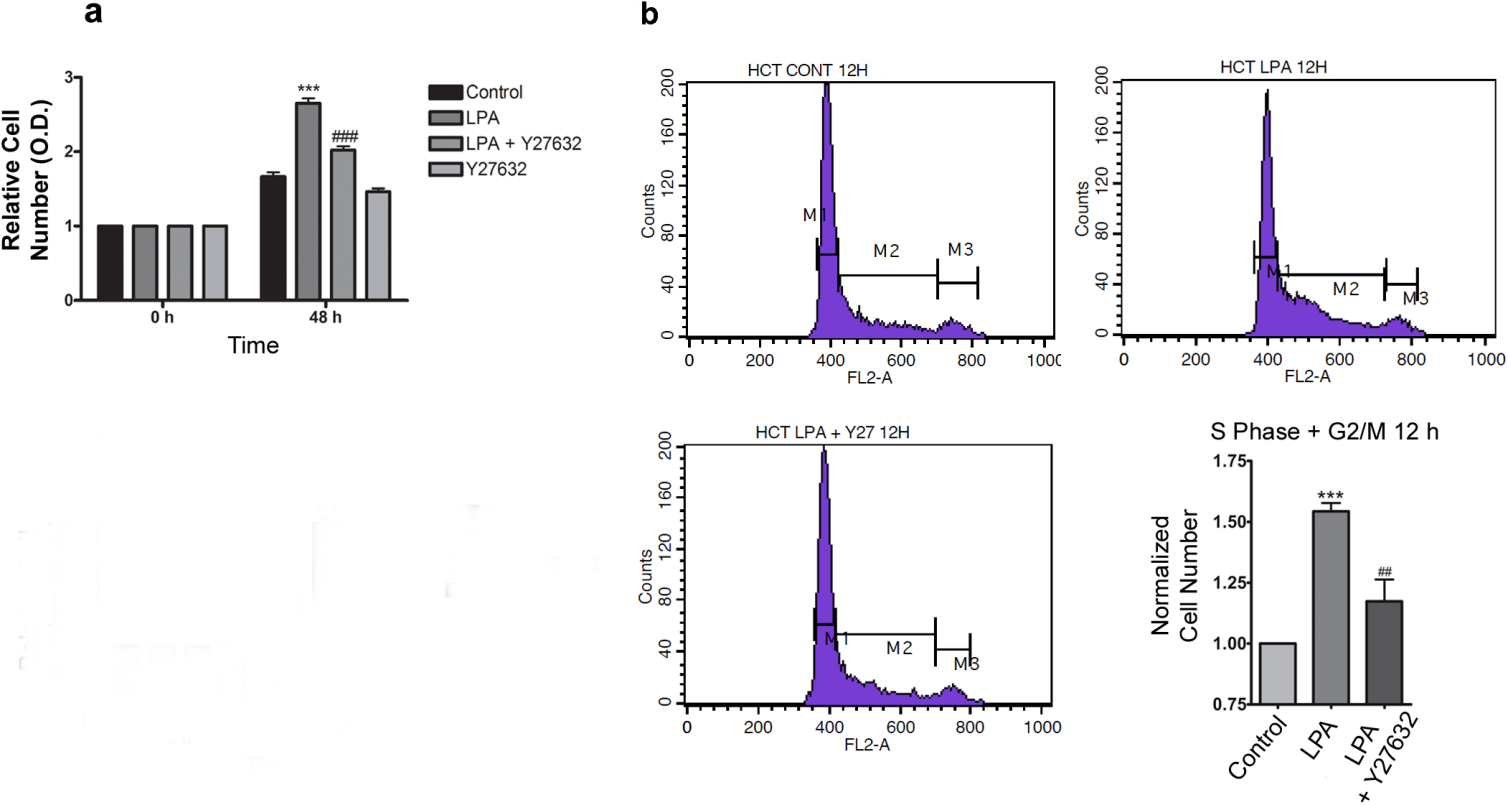
LPA mediates cell proliferation through RhoA-ROCK activation. Subconfluent cell monolayers were FBS depleted for 24 h and treated with LPA at the indicated times. a) Crystal violet staining of HCT-116 showed that the ROCK inhibitor Y-27632 (10 μM) prevented an LPA-mediated increase in the relative cell number after 48 h of treatment. Statistical analyses were performed using *two-way* ANOVA with *post-hoc* Bonferroni test. *** p<0.001, vs control; ### p<0.001, vs LPA. Average scores± SEM. for three independent experiments are shown. b) The FACS analysis via PI staining showed that ROCK inhibition with Y-27632 prevented the LPA-induced increase in the proportion of cells in the S-G2/M phase. The statistical analyses were performed using *one-way* ANOVA with *post-hoc* Bonferroni test. Data are presented as mean ± SEM. (*** p<0.001, vs control; ## p<0.01, vs LPA).

Next, we examined whether LPA activates ROCK downstream of Rho and whether this signaling pathway is responsible for modulating cell proliferation. Thus, we performed a crystal violet assay and cell cycle analysis after the inhibition of ROCK with Y-27632. The results indicate that ROCK inhibition prevented the increase in the relative cell number after treatment with LPA for 48 h (**Fig 4A**). Moreover, the ROCK inhibitor prevented LPA-induced cell cycle progression (**Fig 4B**). These data support the notion that LPA induces HCT-116 cell proliferation through Rho-ROCK activation.

### LPA activates STAT-3 to mediate cell proliferation in a Rho-ROCK independent manner

**Fig 5:**
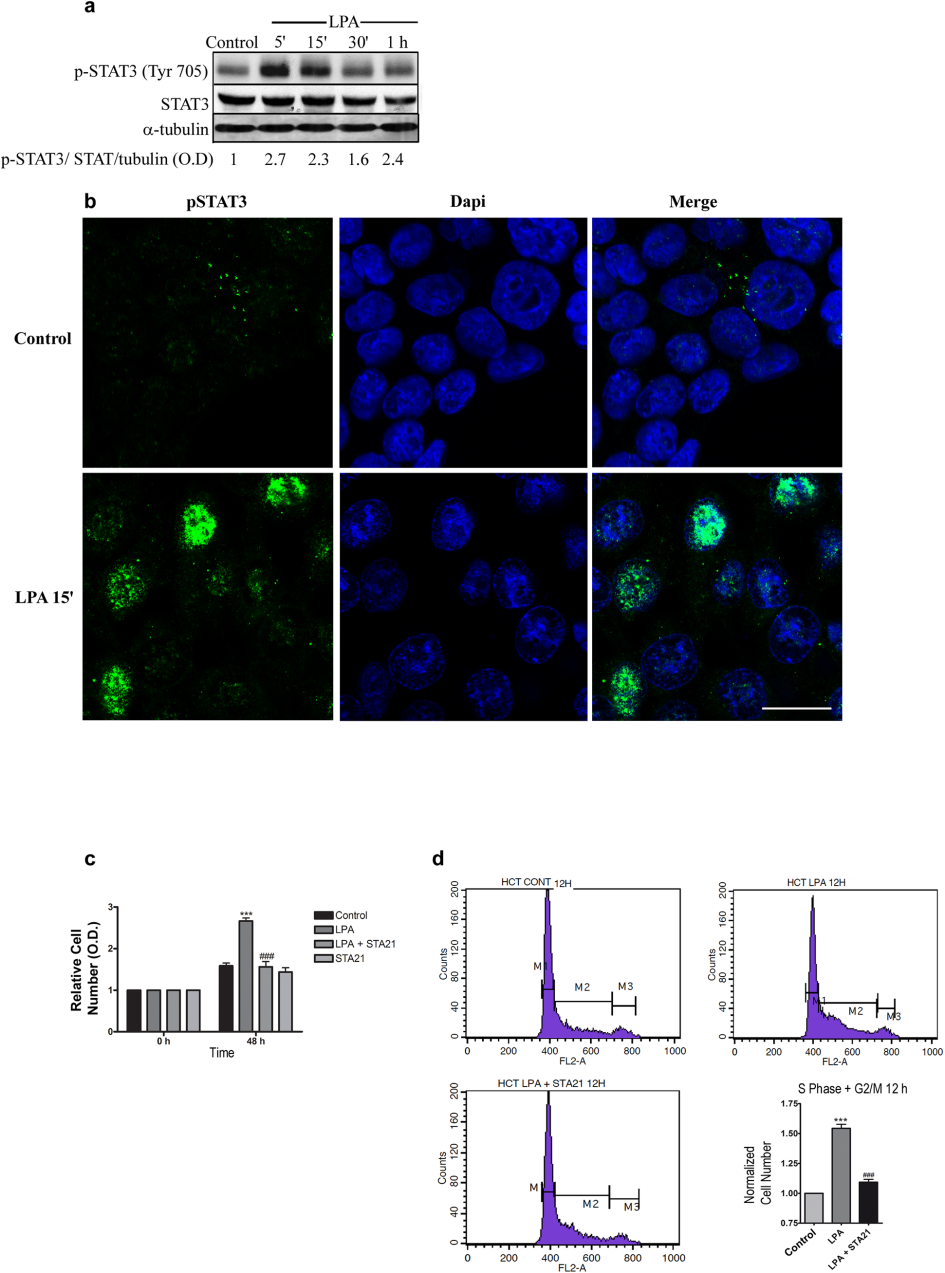
LPA mediates cell proliferation through STAT-3 activation. Subconfluent monolayers of HCT-116 cells were FBS depleted overnight and treated with LPA at the indicated times. a) Total lysates were obtained and prepared for western blotting using a specific antibody against the phosphorylated form of STAT-3 at tyrosine 705 (p-STAT3). The band images were quantified by optical density, and the score was calculated using the ratio between p-STAT3, STAT3 and α-tubulin. LPA increased STAT-3 phosphorylation after 5–15 min of treatment. b) Immunofluorescence of HCT-116 cells corroborated the increase in pSTAT-3 after LPA treatment (green, arrows) and displayed its nuclear location (nucleus, blue). Scale bar, 20 μm. c) The relative cell number of LPA-treated HCT-116 cells was evaluated using crystal violet staining. STAT-3 inhibition using STA 21 prevented the increase in cell numbers after 48 h of LPA treatment. Statistical analyses were performed using *two-way* ANOVA with *post-hoc* Bonferroni test (*** p<0.001, vs control; ### p<0.001, vs. LPA). d) Cell cycle analyses through PI staining indicated that STAT-3 inhibition reduced the number of cells at the S-G2/M phase after LPA treatment. Statistical analyses were performed using *one-way* ANOVA (*** p<0.001, vs. control; ### p<0.001, vs. LPA). Data are presented as mean ± SEM.

It is well known that both LPA and Rho can trigger different signaling pathways to modulate cell proliferation. Moreover, we have previously shown that LPA disrupts adherens junctions in Caco-2 cells through Rho-ROCK signaling [10], and a study performed by Yang *et al*. [8] indicated that LPA activates ß-catenin signaling in HCT-116 cells. Thus, we decided to investigate Wnt/ß-catenin signaling after LPA treatment in HCT-116 cells. Although we have found that LPA increases ß-catenin expression and the phosphorylation of GSK3ß (**Figures a and b in S5 Fig**, respectively), we were not able to detect either the transcriptional activity of ß-catenin using the TCF/LEF reporter assay or the nuclear location of this protein using confocal microscopy (**Figures c and d in S5 Fig**, respectively). Because LPA activates STAT-3 in ovarian cancer cells [22], we decided to evaluate the participation of this transcription factor in our study model. Our biochemical analysis showed that LPA stimulates tyrosine phosphorylation at residue Tyr 705 of STAT-3 for 5–30 min of treatment (**Fig 5A**). To confirm this finding, we performed an immunofluorescence analysis and evaluated the cellular location of activated STAT-3 after 15 min of LPA treatment (**Fig 5B**). Confocal images showed that there is a weak and dispersed staining of pSTAT-3 in the control group, whereas an evident intense nuclear staining of STAT-3 can be observed after LPA treatment. Next, we examined the participation of STAT-3 in LPA-induced HCT-116 proliferation by using STA-21, a pharmacological inhibitor that impairs STAT-3 dimerization and nuclear translocation and consequently STAT-3 activity. The proliferation assay and cell cycle analysis indicated that STAT-3 inhibition prevented the increase in the relative cell number after treatment with LPA for 48 h (**Fig 5C and 5D**, respectively).

**Fig 6:**
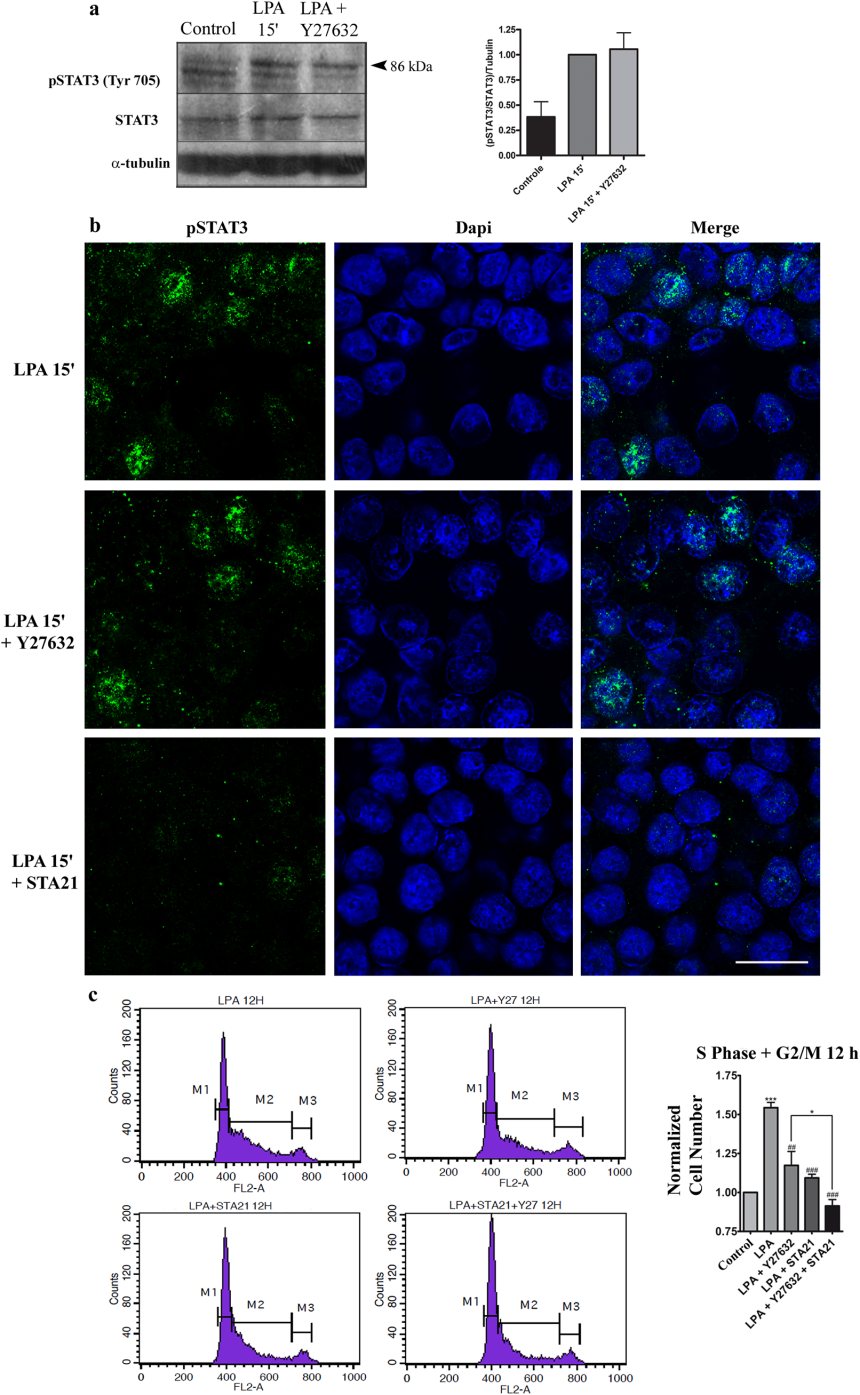
RhoA-ROCK and STAT-3 signaling pathways cooperate to control LPA-mediated cell proliferation. HCT-116 cells were pre-treated with the ROCK inhibitor Y-27632 for 1 h and then treated with LPA as indicated. a) Western blotting against the phosphorylated form of STAT-3 at tyrosine 705 showed that ROCK inhibition did not prevent STAT-3 phosphorylation mediated by LPA. O.D., optical density. Data are presented as mean ± SEM. b) Confocal images indicating that ROCK inhibition did not prevent the LPA induction of pSTAT3 (green) translocation to the nucleus (blue). The STAT-3 inhibitor, STA 21, was used as a positive control. Cont: control. Scale bar, 20 μm. c) Cell cycle analyses through PI staining indicated that the dual inhibition of STAT-3 and ROCK using both STA 21 and Y-27632 significantly reduced the number of cells in the S-G2/M phase after LPA treatment. Statistical analyses were performed using *one-way* ANOVA. Average scores ± SEM. for three independent experiments are shown (*** p<0.001, vs. control; ### p<0.001; ## p<0.01, vs. LPA; * p<0.05, LPA+Y-27632+STA21 vs. LPA + Y-27632).

To further investigate whether ROCK is upstream to STAT-3 in LPA-induced cell proliferation, we performed western blotting for phosphorylated STAT-3 (Tyr 705) after the treatment of HCT-116 cells with LPA alone or in combination with the ROCK inhibitor Y-27632. We found that ROCK inhibition did not prevent STAT-3 activation in LPA-treated cells because Y-27632 did not reduce the tyrosine phosphorylation levels of STAT-3. Likewise, immunofluorescent images obtained by confocal microscopy confirmed that STA-21 indeed impairs the nuclear translocation of STAT-3, yet this inhibitor could not prevent the LPA-mediated nuclear translocation of STAT-3 (**Fig 6B**). Together, these data suggest that LPA activates both the Rho-ROCK and STAT-3 pathways but in an independent manner. We further investigated this hypothesis by treating HCT-116 cells with both the Y-27632 and STA-21 inhibitors simultaneously; we also evaluated the cell cycle by flow cytometry. As we previously showed in **Figs 4B and 5D**, both ROCK and STAT-3 inhibition prevented LPA-induced cell cycle progression from the G1 phase to the S-G2/M phases. Interestingly, the simultaneous inhibition of these proteins prevented cell cycle progression even more than isolated inhibition (**Fig 6C**). This result confirms the finding that Rho-ROCK and STAT-3 cooperate to mediate the LPA-induced proliferation of HCT-116 cells.

### LPA regulates the expression of genes related to the cell cycle

To analyze the alterations in transcriptome expression levels associated with LPA treatment, a global gene expression profiling was performed using HCT-116 cells treated with LPA for 12h, which was the time LPA mediated cell cycle entry, compared to untreated cells. Interestingly, after an *in silico* analysis of these results using MetaCore^TM^ software, we observed that the first two statistically significant pathway maps related to the differentially expressed genes of LPA-treated cells were related to the cell cycle: DNA replication in early the S phase cycle and the role of Adenomatous polyposis coli (*APC*) in cell cycle regulation, respectively (**Figures a and b in S6 Fig**); the third pathway was related to the inhibition of apoptosis (data not shown). After, we analyzed the differentially expressed genes that could be related to the cell cycle, such as cyclins and CDKs/CDCs. As observed in **Table 1,** 17 genes related to the cell cycle were found differentially expressed in treated cells compared to the control cells. Although some differentially expressed genes presented a fold change expression less than 2 (but greater than a 1.2-fold change), it was interesting to observe that all the genes were upregulated in the treated cells to a statistically significant degree (p-value ≤0.05). These results suggested an influence of LPA treatment on cell cycle gene expression. A detailed analysis of all the upregulated and downregulated genes can be observed in **S1** and S**2 Tables**, respectively.

**Table 1:** Upregulated genes related to cell cycle of LPA-treated cells.

Gen	Symbol	Function	Fold *
**Cyclin E2**	CCNE2	Transition G1/S	+ 2.37
**Cell Division cycle 6 homolog**	CDC6	Initiation of DNA replication	+2.08
**Cell Division cycle 45 homolog**	CDC45	Initiation of DNA replication	+ 1.90
**Cyclin A2** **	CCNA2	Transition G1/S and G2/M	+1.87
**Cyclin-dependent Kinase 1**	CDK1	Transition G1/S and G2/M	+1.73
**Cdc28 protein kinase regulatory subunit 1B**	CKS1B	Interacts with CDKs	+1.56
**Cyclin B2**	CCNB2	Transition G2/M	+1.47
**Cell Division cycle 25 homolog A**	CDC25A	Transition G1/S	+1.45
**Cell Division cycle 7 homolog**	CDC7	Transition G1/S	+1.40
**Cell Division cycle 37 homolog**	CDC37L1	Cell Signaling Transduction	+1.35
**Cdc5 Cell Division cycle 5-like**	CDC5L	Transition G2/M	+1.32
**Cyclin T2**	CCNT2	Cell Cycle	+1.32
**Cyclin B1** **	CCNB1	Transition G2/M	+1.28
**Cdc14 Cell Division cycle 14-like homolog A**	CDC14A	Initiation of DNA replication	+1.26
**Cyclin-dependent Kinase 10**	CDK10	G2/M Phase	+1.25
**Cell Division cycle 20 homolog B**	CDC20B	APC activation—Anaphase	+1.24
**Cyclin E1** **	CCNE1	Transition G1/S	+1.23

* Fold changes of LPA-treated cells vs. control groups.

** Genes validated by Western blotting.

**Fig 7:**
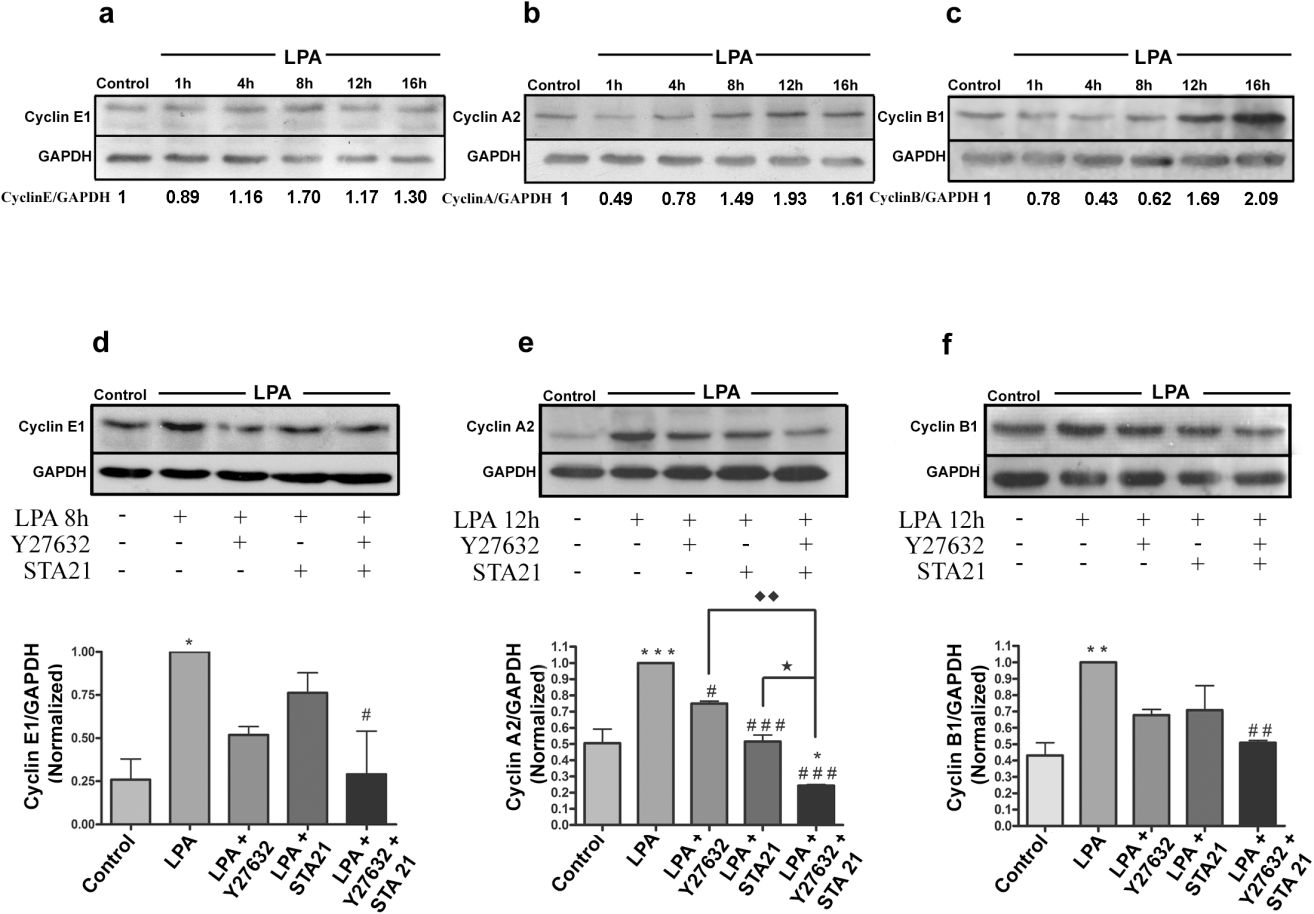
LPA regulates cyclin E1, A2 and B1 expression through Rho-ROCK and STAT-3 signaling. a) HCT-116 cells were treated with LPA at the indicated times, and the total lysates were obtained and prepared for western blotting. LPA increased cyclin E1, A2 and B1 expression. The numbers represent the ratio of the optical density of LPA-treated to untreated cells normalized by GAPDH. b) HCT-116 cells were pre-treated for 1 h with the respective chemical inhibitors prior to LPA treatment at the indicated times. The immunoblotting analysis indicated that either ROCK or STAT-3 inhibition significantly decreased cyclin A2 LPA-mediated expression; however, the concomitant inhibition of these proteins reduced LPA-induced cyclin E1, A2 and B1 expression. The bar graphs are normalized as the percentage of protein expression in which the control group is 1, and the GAPDH protein was used as a loading control. Statistical analyses were performed using *one-way* ANOVA (*** p<0.001, ** p<0.01, * p<0.05, vs. control; ### p<0.001, ## p<0.01, # p<0.05, vs. LPA; ♦♦ p<0.01 vs. LPA+Y-27632; ★ p<0.05 vs.LPA+STA21). O.D., optical density. Average scores± SEM for three independent experiments are shown.

To validate these data, we performed western blotting of the LPA-treated HCT-116 cells. We chose to evaluate cyclins from different cell cycle phases: cyclin E1, which is responsible for the G1-S transition; cyclin A2, which is responsible for the S-G2/M transition; and cyclin B1, which is responsible for the G2-M transition. **Fig 7A–7C** confirms that LPA increases the expression of cyclins E1, A2 and B1 after 8, 12 and 16 h of treatment, respectively. Taken together, we can conclude that LPA increases cell proliferation through inducing cyclin expression, which regulates cell cycle progression. Finally, we verified whether ROCK and STAT-3 participate in this event. Thus, HCT-116 cells were treated with the ROCK inhibitor (Y-27632), the STAT-3 inhibitor (STA21) or both, in the absence or presence of LPA. **Fig 7D–7F** indicates that the simultaneous inhibition of ROCK and STAT-3 prevented the LPA-induced increase in the three analyzed cyclins. It is important to note that this effect was more evident on cyclin A2, which was also regulated by each inhibitor alone. In conclusion, LPA increases cell proliferation through cross-talk between ROCK and STAT-3.

## Discussion

A body of evidence suggests that LPA is a potent inducer of cancer progression at multiple steps; however, there are few studies investigating the role that LPA plays during CRC progression. Different studies have shown that LPA enhances proliferation, survival, and invasion, implying that LPA and its signaling pathways could be potential targets for anti-cancer therapies. Thus, in the present study, we explored the cell transduction mechanisms by which LPA can promote events related to CRC progression.

LPA mediates its biological effects through specific GPCRs. Thus, we initially analyzed the expression of classical LPA receptors (LPA_1-3_) and LPA effects on migration, invasion, survival and proliferation in the CRC cell lines Caco-2, HT-29 and HCT-116. In agreement to the literature [29], we identified a different profile of LPA receptors among these three cell lines (**Fig 1**) and found that the major effect of this biolipid was to increase cell proliferation in HCT-116 cells only (**Fig 2**).

Interestingly, the three cell lines expressed similar levels of LPA_2_, which is related to RhoA activity [27]. Indeed, our results showing that LPA activates RhoA in both Caco-2 [10] and HCT-116 cells (**S4 Fig**) are in agreement with this study. However, it is important to note that RhoA regulates both migration and proliferation [28, 30]. Our results showed that LPA_1_ and LPA_3_ are overexpressed in HT-29 and HCT-116 cells, which are the more invasive cell lines. It was recently shown that LPA_3_ expression in HCT-116 cells inhibits LPA-induced cell migration [16], suggesting that LPA_3_ is a suppressor of cell motility. Likewise, we showed in this study that LPA did not induce cell migration in HT-29 and HCT-116 cells, which express high levels of LPA_3_, whereas the increased cell migration observed in Caco-2 cells could be a result of the low levels of LPA_3_ (**S1 Fig** [10]). The increased cell proliferation in HCT-116 cells in relation to LPA receptor expression is also in agreement with the literature. LPA_2_ and LPA_3_, but not LPA_1_, mediate LPA-induced cell proliferation in HCT-116 and LS173T CRC cells [8]. Moreover, the activation of LPA_1_ promotes the proliferation of DLD1 CRC cells [31]. Together, these data indicate that the biological response to LPA is cell line specific, and the ability of LPA to induce the proliferation of human CRC cells depends on LPA receptor expression. However, further studies are necessary to identify the LPA receptor responsible to mediate cell proliferation in HCT-116 cells.

We then evaluated the mechanisms by which LPA increases cell proliferation in HCT-116 cells. It is well established that LPA activates RhoA, which regulates cell proliferation [14, 30]. As expected, LPA activated RhoA-ROCK signaling pathway to modulate cell proliferation in HCT-116 cells (Fig 4). Other studies have implicated RhoA-ROCK signaling during cell proliferation and support our findings in CRC cells. For instance, the suppression of the RhoA-ROCK pathway has resulted in reduced expression and activities of CDK4 and CDK6, thus inhibiting proliferation and cell cycle G(1)-S transition in gastric cancer lines [32]. Moreover, it was shown that ROCK activation is sufficient to stimulate G1/S cell cycle progression in NIH 3T3 mouse fibroblasts and to alter the levels of cell cycle regulatory proteins such as cyclin D1 and cyclin A [33]. Additionally, members of the Rho family of GTPases are key regulators of the actin cytoskeleton, which regulates the cell cycle through the formation of the actin-myosin contractile ring [28].

The Wnt/ß-catenin signaling pathway is broadly known for its ability to modulate cell proliferation. We have previously shown that LPA disrupts adherens junctions with ß-catenin translocation from cell-cell contacts to the cytosol in Caco-2 cells through Rho-ROCK signaling [10]. Moreover, it was shown that one mechanism by which LPA stimulates the proliferation of colon cancer cells is through the Apc/ß-catenin pathway [8,34]. Therefore, we wanted to determine whether there is cross-talk between RhoA and ß-catenin signaling to mediate proliferation in LPA treated HCT-116 cells. However, we were not able to detect nuclear ß-catenin or TCF/Lef activity in LPA treated cells (**Figures c and d in S5 Fig**). Interestingly, we found that LPA increased ß-catenin expression after 24 h of treatment (**Figure a in S5 Fig**) and indeed inhibited GSK-3β, a Wnt pathway repressor, through its phosphorylation at serine 9 (**Figure b in S5 Fig**). These results suggest that ß-catenin accumulates in response to LPA; however, this appears to be a transitory effect because the expression of this protein is restored after 48 h of LPA treatment. It can be hypothesized that this increase in ß-catenin expression corresponds to a lower affinity β-catenin pool that does not drive transcription and often does not contain a consensus TCF binding motif, as recently described by Schuijers *et al*. [35]. In fact, HCT-116 cells present an oncogenic point mutation in β-catenin in a region of the protein that may be a target for GSK-3β [36]. Previous studies [8,34] showed the involvement of the Wnt/β-catenin in the control of LPA-mediated cell proliferation; however, differences in the experimental procedures could justify the contradictory results between ours and previous studies. We depleted FBS for 24 h and used 10 μM LPA, which activated RhoA (**S4 Fig**). It is important to mention that GSK-3β performs other functions in addition to ß-catenin regulation. For instance, the progressive inactivation of GSK-3β was observed in oral carcinoma, with a positive correlation between the expression of pS^9^GSK-3β and cyclin D1 [37].

The specific dysregulation of STATs has been observed in chronic inflammatory bowel disease [38] and malignant transformation [39, 40]. STAT-3 is activated by phosphorylation at Tyr705, which induces dimerization, nuclear translocation, and DNA binding [41]. Both inactive and active STAT-3 proteins are markedly increased in invasive CRCs. Our findings that LPA-treated cells induced an increase in p-STAT-3 levels and nuclear translocation (**Fig 5A and 5B**, respectively), support the notion that STAT-3 activity is related to CRC progression. The results obtained by immunohistochemistry matching serial sections of normal colonic epithelium and invasive CRCs indicated that nuclear active STAT-3 increased proliferation and lymph node metastasis [42]. Additionally, it was demonstrated that STAT-3 is constitutively activated in CRC cell lines and colorectal specimens [39]. Moreover, it was recently shown that STAT-3 overexpression or STAT-3 activation by IL-6 significantly increased the levels of β-catenin in pancreatic tumor cells, and this effect inhibited β-catenin signaling [43]. Thus, it is possible to hypothesize that STAT-3 activation could also induce an LPA-mediated increase in β-catenin expression with no activation of Wnt signaling in our study model (**S5 Fig**).

Then, we evaluated whether STAT-3 mediates LPA-induced proliferation in HCT-116 cells. The results from **Fig 5C** indicate that STAT-3 inhibition impairs cell proliferation, which is in agreement with a previous study showing that the blockade of JAK3/STAT-3 signaling significantly decreased the viability of colon cancer cells because of apoptosis and cell cycle arrest [44]. To our knowledge, this is the first report showing that LPA activates STAT-3 to regulate cell proliferation.

Next, we wanted to investigate whether there is cross-talk between the Rho-ROCK signaling pathway and STAT-3 in response to LPA in CRC cells. Our results showed that LPA activates both signaling pathways, Rho-ROCK and STAT-3, to mediate CRC cell proliferation (**Figs 4** and **5**). Because ROCK inhibition did not reduce STAT-3 activation or its nuclear translocation in response to LPA (**Fig 6A and 6B**) and because the combined inhibition of ROCK and STAT-3 impaired cell proliferation at higher rates than separate inhibition (**Fig 6C**), we hypothesize that they cooperate to regulate cell cycle control. Furthermore, the fact that the RhoA deletion did not affect the transcriptional activity of STAT-3 in primary fibroblasts [45] supports this idea.

Then, we sought to identify alterations of global gene expression in HCT-116 cells following LPA treatment to determine which genes and signaling pathways involved in CRC progression are affected by LPA. A detailed analysis of the genes with the highest difference in expression showed that many of the factors traditionally associated with the cell cycle were LPA-modulated (**Figures a and b in S6 Fig**). Seventeen genes related to the cell cycle were found to be significantly upregulated in LPA-treated HCT-116 cells compared to control cells (**Table 1**), confirming our previous results. Validation of the microarray data was performed using cyclin E1, which is responsible for the G1-S transition; cyclin A2, which is responsible for the S-G2/M transition; and cyclin B1, which is responsible for the G2-M transition (**Fig 7A–7C)**. The loss of cell cycle checkpoint control is common in CRC. For example, it was shown that CRC samples express high levels of cyclin E, A and D1 [46], and high cyclin B1 expression is a frequent and early event during colorectal carcinogenesis [47].

**Fig 8:**
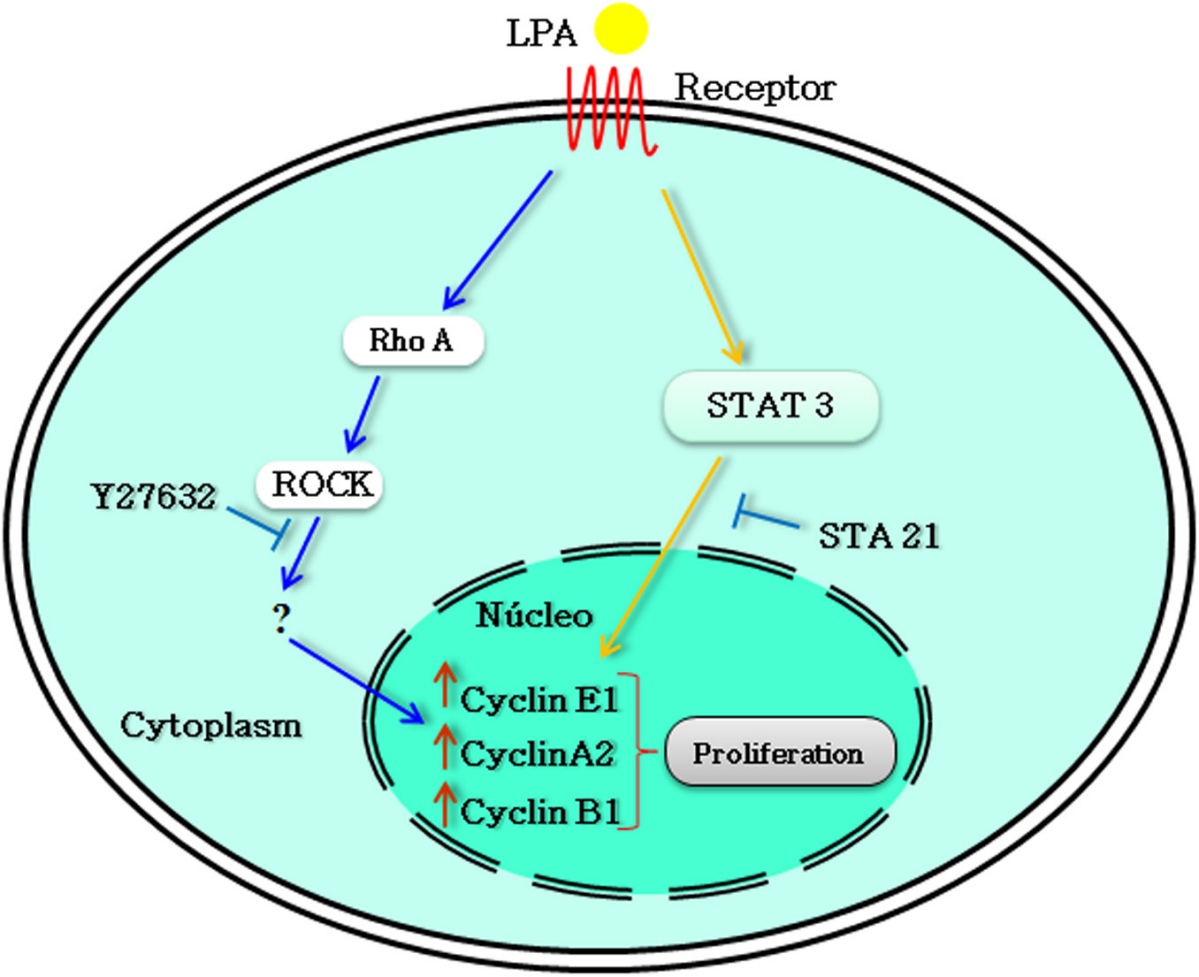
A model for the regulation of the LPA-induced cell cycle. LPA, through its G protein-coupled receptors, induces both Rho-ROCK and STAT-3 signaling pathways to mediate cyclin E1, A2 and B1 expression and to regulate the HCT-116 cell cycle.

Finally, we investigated the role that RhoA-ROCK signaling and STAT-3 play in LPA-mediated HCT-116 cell cycle progression. In **Fig 7D–7F**, we showed that the concomitant inhibition of ROCK and STAT-3 prevented LPA-mediated cyclin overexpression. Moreover, of the analyzed cyclins, only cyclin A2 was significantly prevented from overexpression when ROCK and STAT-3 were separately inhibited. This result agrees with the finding that ROCK activation is sufficient to stimulate G1/S cell cycle progression in mouse fibroblasts by altering the levels of cell cycle regulatory proteins, including cyclin A [33]. Moreover, it was shown that cyclin A2 negatively controls cell motility by promoting RhoA-ROCK activation through a direct interaction with RhoA in breast and colon cancer cells [48]. Whereas many studies have indicated that STAT-3 regulates cyclin D1 [49, 50], some studies suggest that this transcription factor also alters the expression of other cyclins such as cyclin E in pancreatic cancer [51] and cyclin A in our findings. Here, we show that the concomitant inhibition of ROCK and STAT-3 prevented the LPA-mediated increase in cyclin E1, A2 and B1 expression (**Fig 8**).

## Conclusions

In this study we investigate the signaling pathways triggered by LPA to regulate the mechanisms involved in the progression of colorectal cancer cells. We observed that LPA treatment did not affect cell migration, invasion and anchorage-independent growth, but it did induce proliferation and cell cycle progression in HCT-116 cells. Although LPA did not induce transcriptional activity of β-catenin, it promoted the activation of RhoA and STAT-3. Moreover, ROCK and STAT-3 inhibitors prevented LPA-induced proliferation, but ROCK inhibition did not prevent STAT-3 activation. Using the Chip array technique we observed that LPA regulates the expression of genes related to the cell cycle. Finally, the combined inhibition of ROCK and STAT-3 prevented cell cycle progression and increased the LPA-induced expression of cyclins E1, A2 and B1 to a greater degree than either inhibitor alone. Our results demonstrate that LPA increases the proliferative potential of colorectal cancer HCT-116 cells through a mechanism involving cooperation between the Rho-ROCK and STAT3 pathways involved in cell cycle control. Therefore, these two proteins, ROCK and STAT-3, are interesting potential therapeutic targets for the treatment of this cancer type.

## Supporting Information

S1 Fig LPA treatment does not modulate cell migration in HT-29 and HCT-116 cells. Cell migration was evaluated using the wound healing technique. OVCAR-3 (Fig a), HT-29 (Fig b) and HCT-116 (Fig c) cells were serum-starved (control) and incubated with 10 μM LPA for 1 h; cell migration was monitored at 6 and 24 h. The bar graphs display the difference between the two edges of the injury (distance). Each bar represents the mean ± SEM value obtained from three independent experiments. **, p<0.01. A proliferation assay was performed after 24 h of treatment in parallel with the cell migration assay.
(TIF)

S2 Fig LPA does not modulate the invasiveness of colon cancer cells. FBS-depleted Caco-2, HT-29 and HCT-116 cells were plated in Transwell chambers coated with Matrigel^®^, treated with 10 μM LPA for 48 h and subjected to an invasion assay. Fig a) Representative images from three independent experiments. OVCAR-3 cells were used as a positive control of LPA-induced invasiveness. Fig b) The bar graphs display the fold increase of cell invasion (where control = 1). The data are presented as the means ± SEM of triplicate assays for each cell line of three independent experiments. Significance was determined by a one-way ANOVA *** p<0.001.
(TIF)

S3 Fig LPA does not regulate anchorage-independent growth of colon cancer cells. Cells on soft agar plates were grown for 2 weeks in the presence of 10 μM LPA. Fig a) A representative view of each cell line is shown. Fig b) Colony formation was counted and plotted in a normalized graph (control = 1). Significance was determined by a t-test.
(TIF)

S4 Fig LPA activates RhoA in HCT-116 cells. Cells were FBS depleted for 24 h and treated with LPA at the indicated times. Fresh lysates were used to detect the relative amounts of Rho-GTP through a G-LISA assay. LPA increased RhoA activity after 5 and 15 min of treatment. Result of one only experiment.
(TIF)

S5 Fig LPA does not activate Wnt signaling. HCT-116 cells were FBS depleted for 24 h and treated with 10 μM LPA at the indicated times. Cell lysates were obtained and prepared for western blotting against β-catenin (Fig a) and the phosphorylation residue serine 9 from GSK-3β (Fig b). Band images were quantified by optical density (O.D.). LPA increased β-catenin expression and the phosphorylation of GSK-3β. Significance was determined by a t-test; * p<0.05. Fig c) The cells were subjected to a Luciferase Reporter Assay to measure their TCF/LEF activity. The bar graphs display the fold increase in LPA-treated cells of reporter activity compared to control cells in three independent experiments. Significance was determined by a t-test. Average scores± SEM. for three independent experiments are shown. Fig d) The immunoflourescence for β-catenin (green) indicates its predominant location at cell-cell contacts even after LPA treatment. The insets on the superior right section of each panel in the merged images indicate a higher magnification (4X) of the area marked with asterisks. Nucleus (blue); scale bar, 20 μm.
(TIF)

S6 Fig Canonical pathway analyses using MetaCore^TM^ software identified the top two scored pathway maps related to the cell cycle in LPA treated cells. All the maps were drawn from scratch by GeneGo annotators and manually curated and edited. Experimental data are visualized on the maps as blue (for downregulation) and red (upregulation) histograms. The height of the histogram corresponds to the relative expression value for a particular gene/protein (MetaCore™). Fig a) Cell cycle: beginning of DNA replication early in the S phase; Fig b) cell cycle: role of APC in cell cycle regulation. Red thermometers show an object that is upregulated by LPA. Blue thermometers show the objects downregulated by LPA. The large arrow indicates the “pathway start.” TR: transcriptional regulation; CS: complex subunit; B: binding; grey arrow: technical link; green arrows: positive effect; blue arrows: positive interactions; red arrows: negative interactions; grey arrows: unspecified interactions. The boxes on the lines denote the type of regulation: P is phosphorylation, B is binding, and TR is transcriptional regulation.
(TIF)

S1 Table Upregulated genes modulated by LPA treatment (DOC)

S2 Table Downregulated genes modulated by LPA treatment (DOC)
