# Supplementary figures and images for: LPA Induces Colon Cancer Cell Proliferation through a Cooperation between the ROCK and STAT-3 Pathways
Source: PLoS One. 2015 Sep 29;10(9):e0139094. doi: 10.1371/journal.pone.0139094 (PMC4587977; doi:10.1371/journal.pone.0139094)

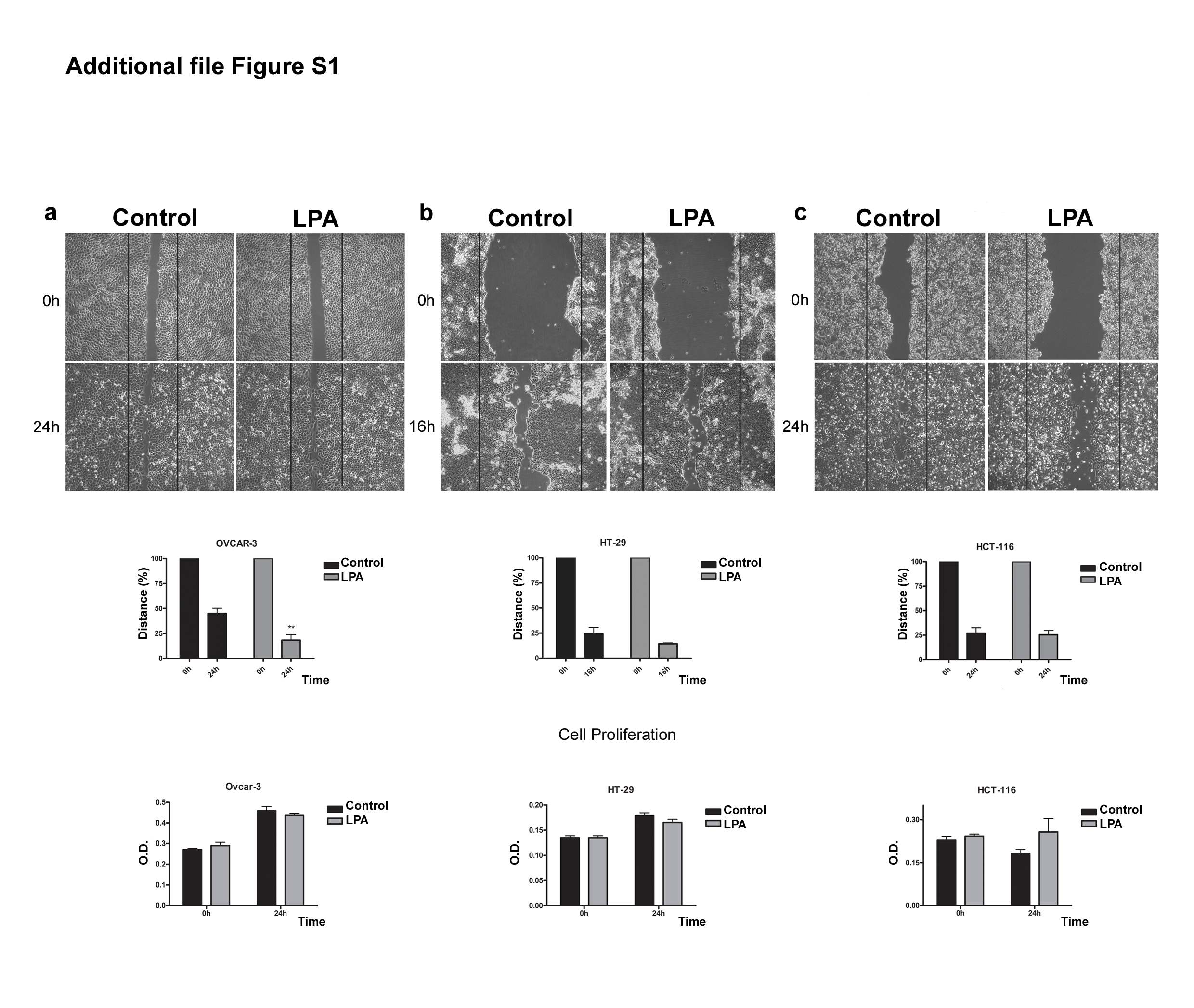

Supplement: S1 Fig — Cell migration was evaluated using the wound healing technique. OVCAR-3 (Fig a), HT-29 (Fig b) and HCT-116 (Fig c) cells were serum-starved (control) and incubated with 10 μM LPA for 1 h; cell migration was monitored at 6 and 24 h. The bar graphs display the difference between the two edges of the injury (distance). Each bar represents the mean ± SEM value obtained from three independent experiments. **, p<0.01. A proliferation assay was performed after 24 h of treatment in parallel with the cell migration assay. (TIF) [file pone.0139094.s001.tif]

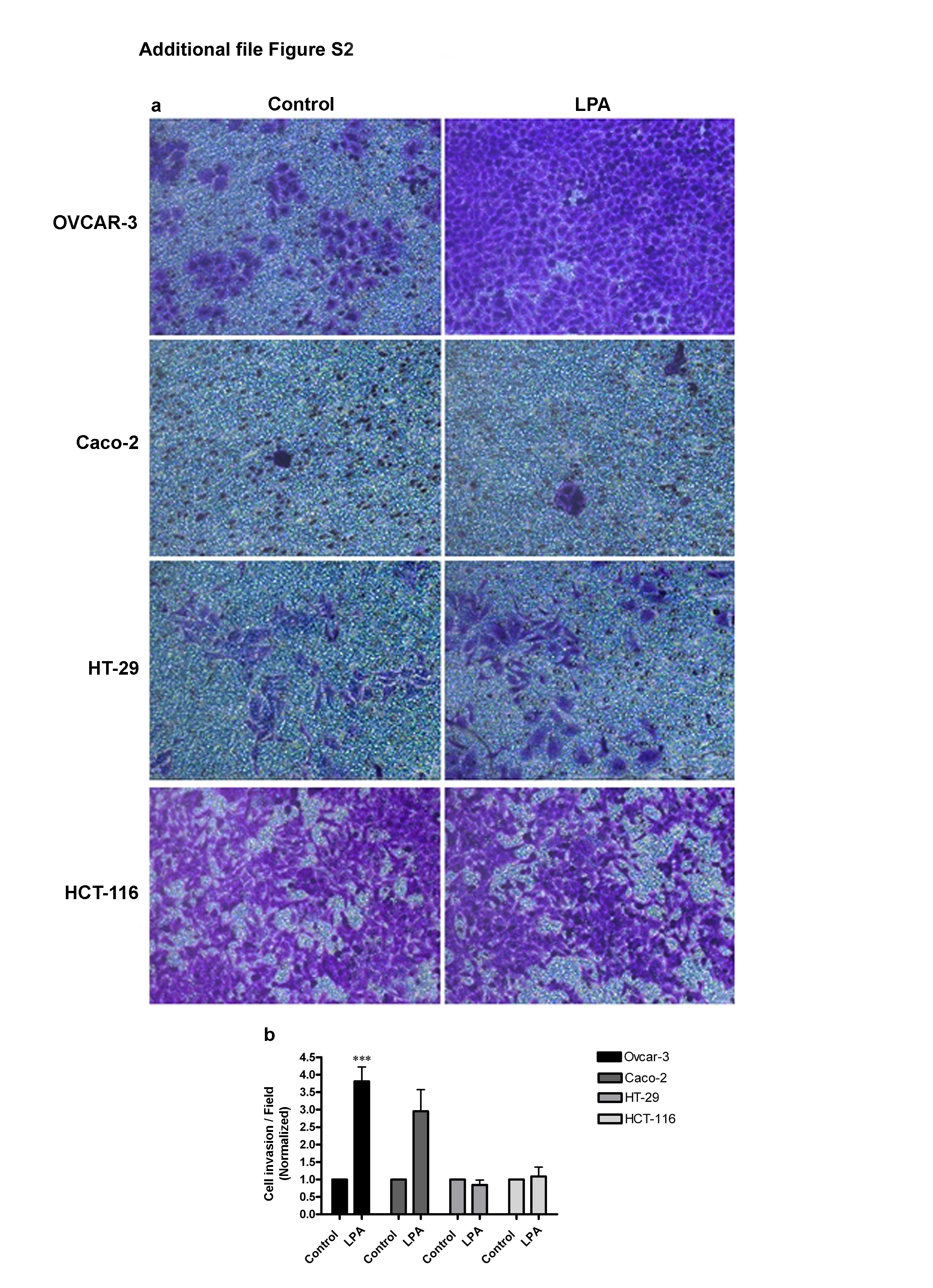

Supplement: S2 Fig — FBS-depleted Caco-2, HT-29 and HCT-116 cells were plated in Transwell chambers coated with Matrigel®, treated with 10 μM LPA for 48 h and subjected to an invasion assay. Fig a) Representative images from three independent experiments. OVCAR-3 cells were used as a positive control of LPA-induced invasiveness. Fig b) The bar graphs display the fold increase of cell invasion (where control = 1). The data are presented as the means ± SEM of triplicate assays for each cell line of three independent experiments. Significance was determined by a one-way ANOVA *** p<0.001. (TIF) [file pone.0139094.s002.tif]

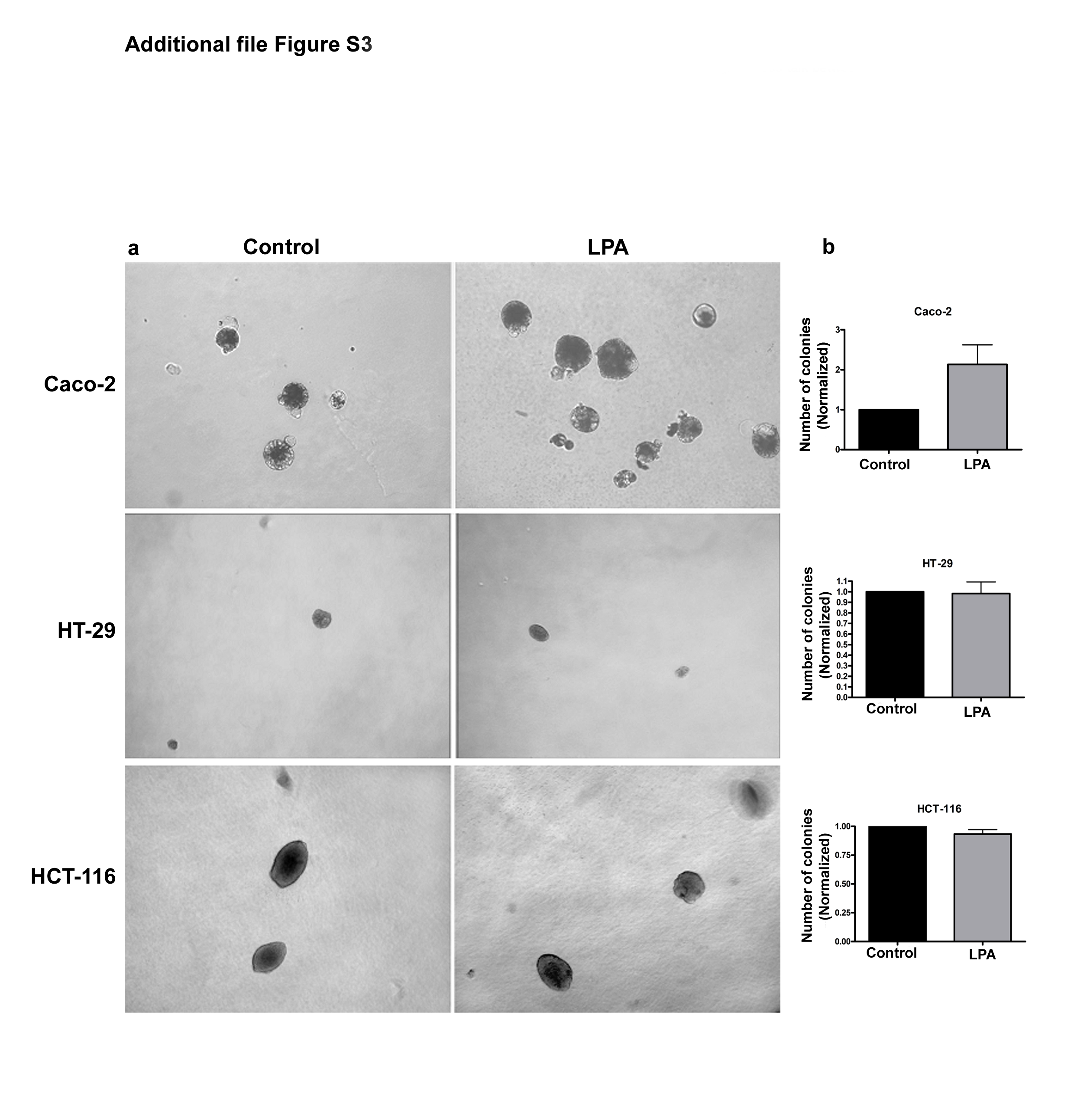

Supplement: S3 Fig — Cells on soft agar plates were grown for 2 weeks in the presence of 10 μM LPA. Fig a) A representative view of each cell line is shown. Fig b) Colony formation was counted and plotted in a normalized graph (control = 1). Significance was determined by a t-test. (TIF) [file pone.0139094.s003.tif]

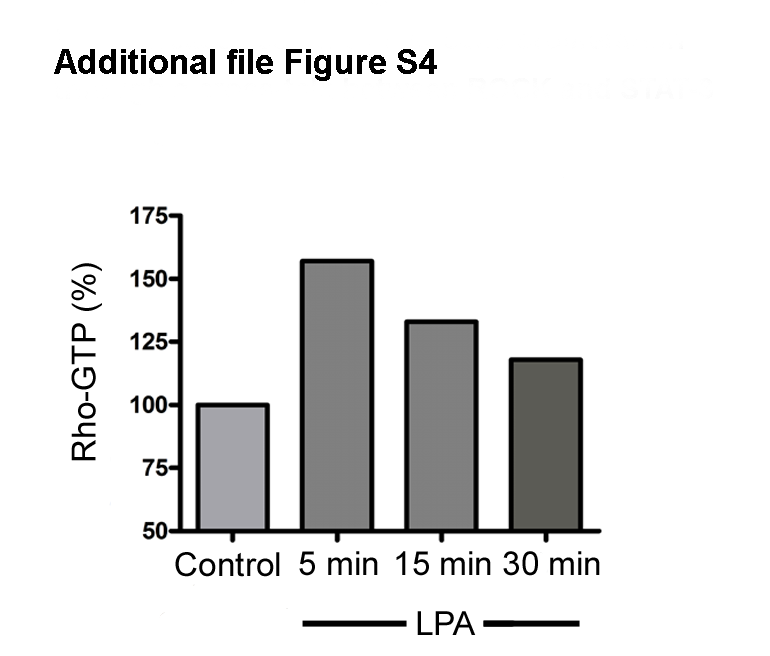

Supplement: S4 Fig — Cells were FBS depleted for 24 h and treated with LPA at the indicated times. Fresh lysates were used to detect the relative amounts of Rho-GTP through a G-LISA assay. LPA increased RhoA activity after 5 and 15 min of treatment. Result of one only experiment. (TIF) [file pone.0139094.s004.tif]

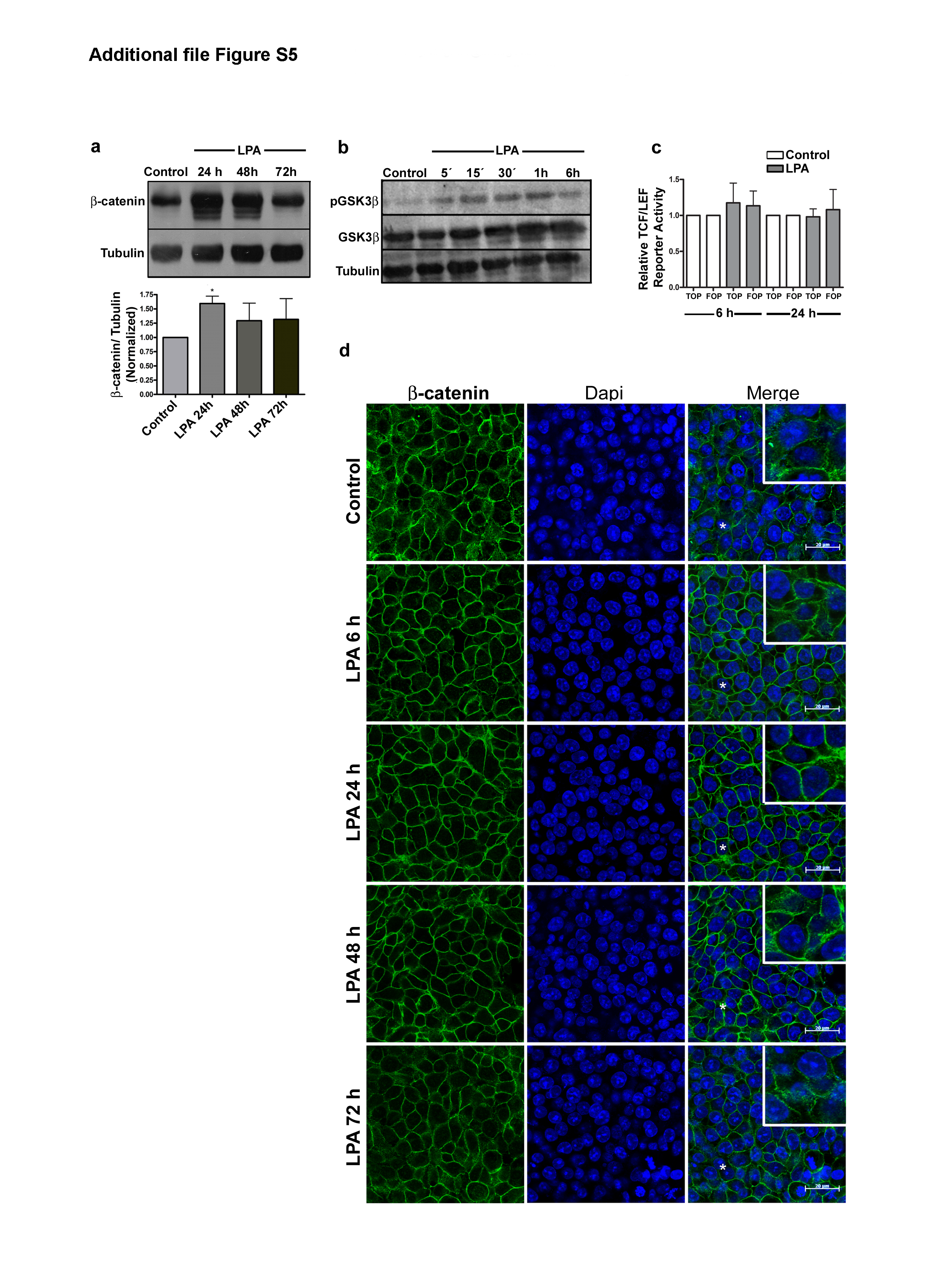

Supplement: S5 Fig — HCT-116 cells were FBS depleted for 24 h and treated with 10 μM LPA at the indicated times. Cell lysates were obtained and prepared for western blotting against β-catenin (Fig a) and the phosphorylation residue serine 9 from GSK-3β (Fig b). Band images were quantified by optical density (O.D.). LPA increased β-catenin expression and the phosphorylation of GSK-3β. Significance was determined by a t-test; * p<0.05. Fig c) The cells were subjected to a Luciferase Reporter Assay to measure their TCF/LEF activity. The bar graphs display the fold increase in LPA-treated cells of reporter activity compared to control cells in three independent experiments. Significance was determined by a t-test. Average scores± SEM. for three independent experiments are shown. Fig d) The immunoflourescence for β-catenin (green) indicates its predominant location at cell-cell contacts even after LPA treatment. The insets on the superior right section of each panel in the merged images indicate a higher magnification (4X) of the area marked with asterisks. Nucleus (blue); scale bar, 20 μm. (TIF) [file pone.0139094.s005.tif]

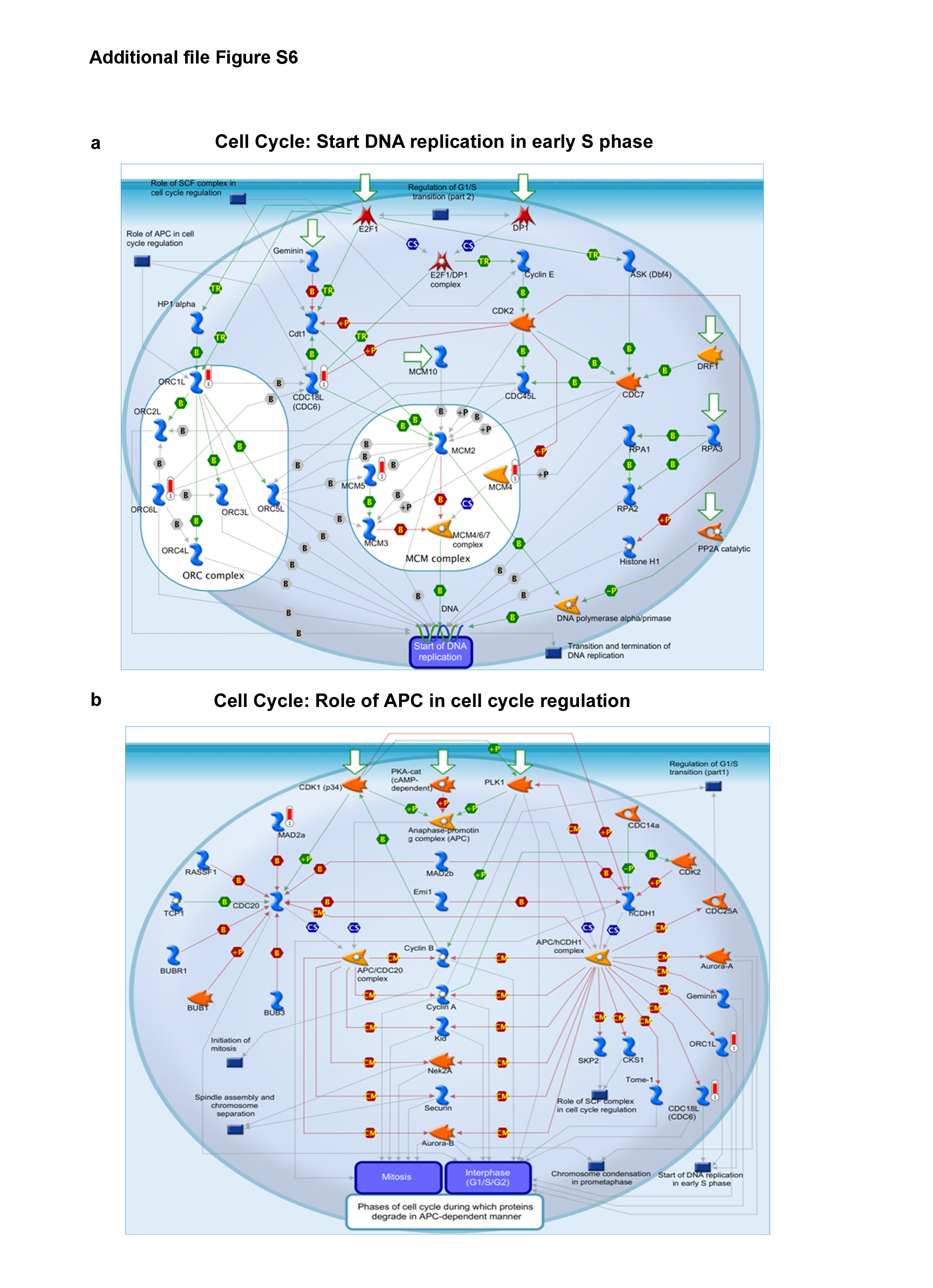

Supplement: S6 Fig — All the maps were drawn from scratch by GeneGo annotators and manually curated and edited. Experimental data are visualized on the maps as blue (for downregulation) and red (upregulation) histograms. The height of the histogram corresponds to the relative expression value for a particular gene/protein (MetaCore™). Fig a) Cell cycle: beginning of DNA replication early in the S phase; Fig b) cell cycle: role of APC in cell cycle regulation. Red thermometers show an object that is upregulated by LPA. Blue thermometers show the objects downregulated by LPA. The large arrow indicates the “pathway start.” TR: transcriptional regulation; CS: complex subunit; B: binding; grey arrow: technical link; green arrows: positive effect; blue arrows: positive interactions; red arrows: negative interactions; grey arrows: unspecified interactions. The boxes on the lines denote the type of regulation: P is phosphorylation, B is binding, and TR is transcriptional regulation. (TIF) [file pone.0139094.s006.tif]
