# Supplementary material for: LPA Induces Colon Cancer Cell Proliferation through a Cooperation between the ROCK and STAT-3 Pathways
Source: PLoS One. 2015 Sep 29;10(9):e0139094. doi: 10.1371/journal.pone.0139094 (PMC4587977; doi:10.1371/journal.pone.0139094)
Supplement: S1 Table — (DOC) [file pone.0139094.s007.doc]

**Additional File Table S1. Upregulated genes modulated by LPA treatment**

| **Gene Symbol** | **RefSeq** | **Fold change (LPA vs. Control)** |
| --- | --- | --- |
| **SNORD115-11** | NR_003303 | 6.47 |
| **SNORD115-26** | NR_003343 | 5.70 |
| **SNORD115-12** | NR_003304 | 5.55 |
| **USP17L6P** | NR_027279 | 4.79 |
| **SNORD115-42** | NR_003357 | 4.34 |
| **SNORD115-20** | NR_003312 | 4.30 |
| **SNORD115-6** | NR_003298 | 3.90 |
| **SNORD115-1** | NR_001291 | 3.65 |
| **SNORD115-44** | NR_003359 | 3.16 |
| **USP17L2** | NM_201402 | 2.88 |
| **RFC1** | L23320 | 2.82 |
| **REXO1L1** | AF495523 | 2.74 |
| **GK3P** | NR_026575 | 2.66 |
| **SNORD115-17** | NR_003309 | 2.61 |
| **ORC6** | NM_014321 | 2.55 |
| **USP17** | NM_001105662 | 2.54 |
| **ADI1** | NM_018269 | 2.51 |
| **FUNDC2** | NM_023934 | 2.51 |
| **LOC100288884** | AK124122 | 2.50 |
| **DTL** | NM_016448 | 2.43 |
| **FAM111B** | NM_198947 | 2.41 |
| **CCNE2** | NM_057749 | 2.38 |
| **SNORD116-15** | NR_003330 | 2.33 |
| **MCM5** | NM_006739 | 2.33 |
| **DUSP5P** | AK055963 | 2.32 |
| **REXO1L2P** | NR_003594 | 2.31 |
| **CENPQ** | NM_018132 | 2.31 |
| **HIST1H2AB** | NM_003513 | 2.29 |
| **LOC100508181** | XM_003120300 | 2.29 |

| **ACAT2** | NM_005891 | 2.29 |
| --- | --- | --- |
| **LOC349196** | NR_027000 | 2.28 |
| **IGKC** | BC073772 | 2.24 |
| **GPAM** | AK172782 | 2.17 |
| **CLSPN** | NM_022111 | 2.17 |
| **HSD17B7P2** | NR_003086 | 2.17 |
| **ZBTB2** | NM_020861 | 2.15 |
| **MAD2L1** | NM_002358 | 2.14 |
| **PSMC3IP** | NM_016556 | 2.14 |
| **SKA3** | NM_145061 | 2.12 |
| **DSCC1** | NM_024094 | 2.11 |
| **ORC1** | NM_004153 | 2.10 |
| **RBM8A** | BC017770 | 2.09 |
| **CDC6** | NM_001254 | 2.09 |
| **FANCI** | NM_001113378 | 2.08 |
| **RRM2** | NM_001165931 | 2.07 |
| **MND1** | NM_032117 | 2.06 |
| **MCM4** | NM_005914 | 2.05 |
| **GINS2** | NM_016095 | 2.04 |
| **NMI** | NM_004688 | 2.03 |
| **TRIP13** | NM_004237 | 2.02 |
| **MARVELD2** | NM_001038603 | 2.02 |
| **HIST1H2BM** | NM_003521 | 2.01 |
| **EXO1** | NM_130398 | 2.01 |
| **NSMAF** | NM_003580 | 2.00 |
