# Supplementary material for: LPA Induces Colon Cancer Cell Proliferation through a Cooperation between the ROCK and STAT-3 Pathways
Source: PLoS One. 2015 Sep 29;10(9):e0139094. doi: 10.1371/journal.pone.0139094 (PMC4587977; doi:10.1371/journal.pone.0139094)
Supplement: S2 Table — (DOC) [file pone.0139094.s008.doc]

**Additional File Table S2. Downregulated genes modulated by LPA treatment**

| **Gene Symbol** | **RefSeq** | **F Fold change (LPA vs. Control)** |
| --- | --- | --- |
| **NGFR** | NM_002507 | -2,89 |
| **SESN3** | NM_144665 | -2,88 |
| **LOC100130876** | AK130278 | -2,81 |
| **OR51I1** | NM_001005288 | -2,75 |
| **TMEM14E** | NM_001123228 | -2,69 |
| **TCP11L2** | NM_152772 | -2,67 |
| **TAS2R4** | NM_016944 | -2,64 |
| **MAP1B** | NM_005909 | -2,63 |
| **RASSF2** | NM_014737 | -2,52 |
| **EGR1** | NM_001964 | -2,46 |
| **SLC29A4** | NM_001040661 | -2,40 |
| **OR51J1** | ENST00000332043 | -2,39 |
| **FAM131C** | NM_182623 | -2,34 |
| **SNORD13P2** | X58060 | -2,33 |
| **ST3GAL5** | NM_003896 | -2,28 |
| **STAT3** | NM_139276 | -2,24 |
| **LOC100127886** | AF090938 | -2,24 |
| **C17orf61** | BC030270 | -2,23 |
| **SAT1** | NR_027783 | -2,23 |
| **SH3BGRL2** | NM_031469 | -2,22 |
| **NDRG1** | NM_001135242 | -2,22 |
| **RCAN2** | NM_005822 | -2,17 |
| **AHNAK2** | NM_138420 | -2,17 |
| **RNU1-1** | NR_004430 | -2,17 |
| **SNORA64** | NR_002326 | -2,15 |
| **SPARC** | NM_003118 | -2,12 |
| **LEMD1** | NM_001001552 | -2,12 |
| **ARSI** | NM_001012301 | -2,12 |
| **UTRN** | NM_007124 | -2,10 |
| **GLRX** | NM_002064 | -2,10 |
| **GM2A** | NM_000405 | -2,10 |
| **ARRDC4** | NM_183376 | -2,10 |
| **C6orf99** | NM_001195032 | -2,09 |
| **LOC440993** | AK128346 | -2,09 |
| **SGPP2** | NM_152386 | -2,08 |
| **NOTCH3** | NM_000435 | -2,07 |
| **CECR2** | NM_031413 | -2,06 |
| **MIR181A2** | NR_029611 | -2,06 |
| **GPR183** | NM_004951 | -2,06 |
| **NBEA** | NM_015678 | -2,06 |
| **LIFR** | NM_002310 | -2,06 |
| **C7orf54** | NR_027330 | -2,05 |
| **OR51M1** | NM_001004756 | -2,05 |
| **ANO4** | NM_178826 | -2,04 |
| **SNORA23** | NR_002962 | -2,04 |
| **BBS9** | NM_198428 | -2,03 |
| **ANXA6** | NM_001155 | -2,03 |
| **FAM114A1** | NM_138389 | -2,02 |
| **TSHZ2** | NM_173485 | -2,01 |
